# Light‐Imprinted Chirality in Nanomaterials: From Principles to Applications

**DOI:** 10.1002/advs.202524331

**Published:** 2026-07-30

**Authors:** Xinru Jin, Emiliano Cortés, Alexander O. Govorov, Tao Ding

**Affiliations:** ^1^ Key Laboratory of Artificial Micro/Nano Structure of Ministry of Education School of Physics and Technology Wuhan University Wuhan People's Republic of China; ^2^ Nanoinstitute Munich, Faculty of Physics Ludwig‐Maximilians‐Universität (LMU) Munich Germany; ^3^ Department of Physics and Astronomy Ohio University Athens Ohio USA

**Keywords:** circularly polarized light, hot electrons, light‐induced chirality, optical vortex, tunable chirality

## Abstract

Light‐induced chirality represents a transformative paradigm for fabricating chiral nanostructures, offering unique advantages over traditional approaches including chemical synthesis, self‐assembly, and lithography. This review provides a comprehensive framework encompassing light‐based strategies for imprinting and tuning chirality in nanomaterials, which includes laser direct writing, optical manipulation, CPL‐induced symmetry breaking with the assistance of surface plasmons, optical vortex structuring, and momentum transfer. We critically compare different optical approaches, providing clear selection guidelines based on resolution, scalability, material compatibility, and equipment requirements. We also examine dynamic light‐modulated chirality for switchable and reconfigurable structures, and highlight their emerging applications in biomedicine, sensing, polarization optics, and chiral displays. Finally, we conclude this review with current challenges and future directions. By establishing light‐induced chirality as the most promising route for scalable chiral nanofabrication, this review guides researchers in harnessing light to create next‐generation functional materials.

## Graphical abstract

Light‐induced chirality represents a transformative paradigm for fabricating chiral nanostructures. This review provides a comprehensive framework encompassing light‐based strategies for imprinting and tuning chirality in nanomaterials, which guides researchers in harnessing light to create next‐generation functional materials.

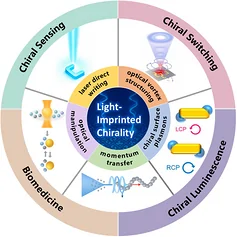

## 1 Introduction

Chirality, the property of objects that cannot be superimposed on their mirror images, plays a fundamental role in nature from molecular biology to cosmology [1]. In the realm of nanotechnology, chirality at the nanoscale has emerged as a critical design parameter for advanced materials due to their ability to manipulate circularly polarized light (CPL) at the nanoscale, leading to enhanced circular dichroism (CD) and optical rotation far exceeding those of natural chiral molecules [2]. These extraordinary chiroptic properties arise from the coupling between the structural chirality and photonic resonances, creating opportunities for ultrasensitive chiral sensing, enantioselective catalysis, negative refractive index materials, and advanced optical information processing [3, 4, 5].

The fabrication of chiral photonic nanostructures presents unique challenges that distinguish it from conventional nanofabrication. Beyond achieving nanoscale precision, these methods must introduce and control three‐dimensional (3D) chirality while maintaining the plasmonic properties of the constituent materials. Over the past decade, numerous fabrication strategies have been developed, each with distinct advantages and limitations [6, 7, 8, 9, 10, 11]. However, a critical gap has emerged between the ability to create individual chiral nanostructures with excellent properties and the need to organize them into functional devices requiring regular, addressable arrays.

### 1.1 Fabrication Strategies of Chiral Nanomaterials: Comparative Analysis

Due to their extraordinary chiroptic properties of chiral nanomaterials, significant progress has been made in the fabrication of various kinds of chiral nanostructures through different strategies in the last two decades. Several seminal reviews have touched most of them more than 5 years ago [3, 7, 12, 13, 14, 15, 16, 17, 18, 19], and an updated revisit and comparative analysis is timely important to reveal the new trend of chiral nanofabrication. Conventional strategies of chiral nanofabrication include direct chemical synthesis of chiral nanoparticles (NPs) [20], self‐assembly of achiral NPs into chiral superstructures [21], glancing angle deposition (GLAD) of helical NPs [22], nanolithographic approach toward chiral metasurfaces including electron beam, focused‐ion beam and nanoimprint lithography (EBL/FIB/NIL) [23], and light‐directed chirality such as two‐photon lithography (TPL) and CPL induced chiral growth [24]. These methods have different advantages and disadvantages, and proper selection of fabrication strategy depends on the purpose of the research. For instance, DNA‐origami strategy might be the best for exploring new architectures for fundamental research. Lithographic methods work the best for high‐precision devices. Light‐directed approaches are more suitable for practical devices and metasurfaces. For performance and application‐oriented research, facileness, regularity, precision, scalability, tunability, ligand‐free are the key criteria to evaluate different fabrication methods as they are crucial factors for robust, cost‐effective and high quality chiroptic devices that can sustain their practical applications. In the following section, each fabrication method will be evaluated against the above‐mentioned criteria for comparative analysis.

#### 1.1.1 Chemical Synthesis

**FIGURE 1:**
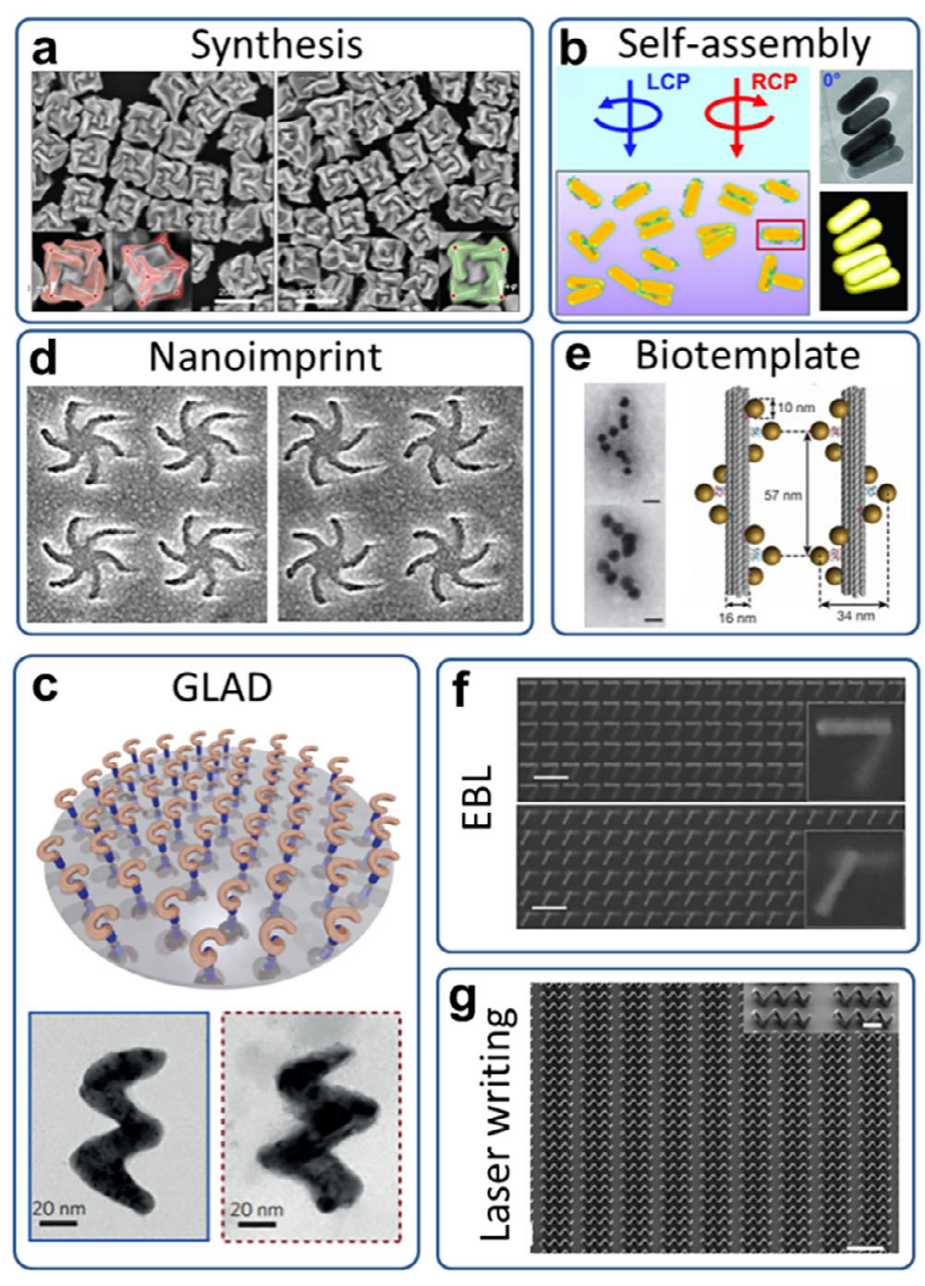
Fabrication strategies of chiral plasmonic nanostructures. (a) Chemically synthesized chiral NPs. SEM images of Au helicoids synthesized with L‐Cysteine (Left) and D‐Cysteine (Right). Insets are marked helicoids with [100]/[111] orientations. Reproduced with permission [25]. Copyright 2018, Springer Nature. (b) Chiral self‐assembly system based on Au nanorod‐BSA complexes. Left: Schematic of the self‐assembly, Right: cryo‐TEM image and reconstruction model. Reproduced with permission [34]. Copyright 2019, American Association for the Advancement of Science. (c) Spiral nanostructures fabricated by GLAD. Top: Schematic of 3D helix formation via substrate angle control; Bottom: TEM images of Ag/Cu alloy helix (left) and Cu helix (right). Reproduced with permission [38]. Copyright 2013, Springer Nature. (d) SEM images of plasmonic shuriken nanostructures fabricated by nanoimprint, featuring right‐ and left‐handed nanoshurikens. Reproduced with permission [42]. Copyright 2015, John Wiley and Sons. (e) Plasmonic helical self‐assembly via DNA‐origami template. Left: TEM images of LH and RH Au NP helices with DNA template (scale bars:20 nm). Right: Schematic of Au NP assembly on DNA‐origami template (Ø 34 nm, pitch 57 nm). Reproduced with permission [35]. Copyright 2012, Springer Nature. (f) SEM of crossed nanobars with +60° (Top) and −60° (Bottom) configuration. Reproduced with permission [43]. Copyright 2017, Springer Nature. (g) Arrays of helical nanowires fabricated by direct laser writing. Inset is magnified view of the helices. Scale bars are 2 µm in the inset and 10 µm for the overview. Reproduced with permission [47]. Copyright 2012 John Wiley and Sons.

Chemical synthesis of chiral NPs mostly involves the use of chiral ligands, which induce chiral growth from an achiral seed to form homochiral NPs. Typical examples include helicoids (Figure 1a), [25] chiral nanorods [26, 27, 28], and nanotriskelion [29]. They can be produced in bulk via standard chemistry but difficult to form exact geometries with well‐defined orientation and arrangements, rendering device‐based application challenging. Its major advantage is batch scalability, because large quantities of chiral NPs can be obtained through standard wet‐chemical protocols. However, this advantage is accompanied by limited control over spatial registration, particle orientation, and array‐level organization. These limitations make direct integration into addressable chiral photonic devices difficult. The tunability of chemically synthesized chiral NPs depends strongly on ligand identity, precursor concentration, ionic environment, and reaction kinetics, which may affect reproducibility across different batches. Moreover, chiral ligands remaining on the particle surface can interfere with surface accessibility and introduce additional molecular chiroptical contributions, complicating the analysis of plasmonic CD or sensing responses. Thus, chemical synthesis is most suitable for scalable colloidal production, whereas deterministic device integration generally requires subsequent assembly, alignment, or patterning.

#### 1.1.2 Self‐Assembly

Self‐assembly represents a bottom‐up approach where components spontaneously organize into ordered structures through non‐covalent interactions. This chiral self‐assembly process could be surfactant‐directed, peptide‐driven, or solvent‐induced, which harnesses intrinsic molecular forces or external fields (like mechanic [30, 31] and magnetic [32] fields) to create chiral architectures [33]. This approach is particularly attractive as it mimics many of the biological systems for fundamental understanding of the chiral original of life [11]. These self‐assembly processes normally occur in solution or at interfaces with the assistance of (bio)molecules such as bovine serum albumin (BSA), which show strong chiroptic response (Figure 1b) [34]. However, their assembly precision and regularity are poor unless an DNA‐origami template is used (Figure 1e) [35]. The unique advantage of using DNA‐origami is their atomic precision with programmable chirality [36]. However, these templates still struggle with scalability, cost, and the critical challenge of organizing individual structures into regular arrays. The reliance on expensive biological scaffolds and limited environmental stability further restrict practical applications. Therefore, self‐assembly offers a powerful route to structurally diverse and bioinspired chiral architectures, especially when the goal is to explore nanoscale organization, collective optical response, or structure‐property relationships. Nevertheless, its device‐level implementation remains constrained by limited positional addressability, incomplete assembly yield, batch‐to‐batch variability, and the difficulty of registering individual chiral units over large areas. Although DNA‐origami templates greatly improve nanoscale precision and chirality programmability, they do not fully solve the problems of scalability, cost, environmental stability, and integration with solid‐state photonic platforms. Moreover, the ligands, surfactants, peptides, or biological scaffolds required for assembly may modify surface chemistry and introduce additional optical or chemical complexity. Therefore, self‐assembly is most suitable for fundamental studies and bioinspired chiral materials, while practical chiral photonic devices usually require subsequent alignment, immobilization, transfer, or lithographic registration.

#### 1.1.3 GLAD

GLAD is a physical vapor deposition technique where material flux arrives at the substrate at highly oblique angles (typically 70°–89° from the normal direction of surface) [37]. Combined with substrate rotation, GLAD creates 3D nanostructures with controlled chirality through self‐shadowing effects (Figure 1c) [38]. It is a ligand‐free and template‐free physical route that can be applied to a broad range of materials, including metals, semiconductors, oxides, and dielectric materials. Its main advantage is the direct formation of three‐dimensional chiral architectures over relatively large substrate areas without relying on molecular ligands or wet‐chemical assembly. This makes GLAD attractive for chiral coatings, polarization optics, and large‐area metamaterial films. However, the final morphology is strongly governed by self‐shadowing, nucleation statistics, deposition angle, rotation rate, and surface diffusion during growth. As a result, GLAD provides less deterministic control over the geometry of individual nanoscale units than lithographic methods. Defect formation, local height variation, and limited positional addressability also make it challenging to integrate GLAD‐grown helices into complex device architectures that require precisely registered unit cells. Therefore, GLAD is most suitable for ligand‐free, large‐area chiral films and coatings, whereas applications requiring arbitrary patterns, nanoscale addressability, or independently programmable chiral meta‐atoms generally require lithographic or optical writing strategies.

#### 1.1.4 Nanolithography (EBL/FIB/NIL)

Top‐down approaches such as EBL and FIB provide excellent precision and array formation with arbitrary patterns but require complicated operation, expensive equipment, cleanroom facilities, and serial processing that limits throughput [39, 40]. These characteristics make EBL and FIB particularly suitable for proof‐of‐concept chiral metasurfaces, deterministic unit‐cell design, and mechanistic studies where geometric accuracy and positional addressability are more important than manufacturing throughput. However, their serial nature and high infrastructure cost remain major obstacles for large‐area production, especially when centimeter‐scale or wafer‐scale chiral optical components are required. The facileness and scalability issues can be addressed by NIL as it first transfers the patterns to a master mold with EBL, which can be used for NIL for many times with planar scalability [41]. It has been applied for disposable plasmonic chiral metasurfaces (Figure 1d) [42], therefore NIL is more appropriate for high‐throughput replication of predesigned planar chiral patterns than for rapidly exploring new geometries or dynamically tuning chiral responses. Its practical value is highest when the target structure can be fixed in a reusable mold and when surface residues introduced during imprinting do not compromise the intended optical or sensing function. Another critical constrain of most lithographic techniques is they are good at making planar structures, which makes the true 3D chirality challenging. Although quasi‐3D metastructures of crossed nanorods or helices can be obtained via layer‐by‐layer EBL (Figure 1f) [43] or superplastic nanomolding [44, 45, 46], the operation complexity significantly increases, which may increase the probability of local defects and errors. Therefore, lithographic methods remain indispensable for deterministic planar and quasi‐3D chiral metasurfaces, but their advantages become less straightforward when the target requires true 3D chirality, rapid structural reconfiguration, or scalable fabrication of volumetric chiral architectures.

#### 1.1.5 Light‐Induced Chirality

Light‐induced chirality utilizes electromagnetic radiation to drive the formation of chiral structures. This can occur through multiple mechanisms, unified by the principle that light carries energy, momentum, and angular momentum that can be transferred to matter. The most typical approach is laser direct writing (LDW)‐based on two‐photon lithography (TPL), which can create arbitrary 3D patterns in photoresist, such as helical arrays (Figure 1g) [47]. Beyond TPL, optical fields can also direct chiral growth, assembly, and reconfiguration through polarization‐dependent photochemistry [48], optical forces [49], plasmonic near fields [50], or angular‐momentum transfer [51]. These strategies are attractive for maskless and non‐contact fabrication, especially when handedness needs to be selected directly by the light field. However, their performance remains constrained by optical resolution, material compatibility, field‐uniformity control, and processing throughput. Thus, light‐induced chirality should be considered a complementary platform for 3D structuring, dynamic reconfiguration, and site‐selective chiral nanofabrication.

The engineering requirements for chiral light control are strongly application‐dependent. For chiral sensing, the key requirements include intense local optical chirality, low background CD, reproducible nanoscale hotspots, and chemical accessibility of the sensing surface. For circularly polarized emission, efficient performance requires spatial and spectral overlap between emitters and chiral optical modes, high luminescence dissymmetry factor, low optical loss, and compatibility with electroluminescent or photoluminescent device architectures. For polarization optics and chiral metasurfaces, large‐area patterning, high transmission, low insertion loss, and stable polarization conversion are essential. For biomedical applications, colloidal stability, biocompatibility, batch scalability, and surface functionalization are more critical than deterministic array addressability. For quantum and single‐photon chiral photonics, emitter placement accuracy, nanoscale mode volume, low loss, and deterministic coupling to chiral optical modes become the dominant constraints.

These different application scenarios impose different physical constraints on fabrication methods. Relevant descriptors include the minimum feature size, accessible structural dimensionality, material scope, fabrication precision, throughput, and tunability are listed in Table 1. Therefore, a useful comparison of fabrication strategies should not rely on a single universal ranking, but should identify which method satisfies which engineering requirement.

**TABLE 1:** Engineering‐oriented comparison of representative chiral nanofabrication strategies.

Method	Feature size	Geometry	Material	Precision	Throughput	Tunability
chemical synthesis	sub‐10 to 100 nm	colloidal particles	metals, semiconductors	low	high	low
self‐assembly	nm to µm	2D/3D assemblies	broad	medium to low	medium	medium
DNA origami	few nm to tens of nm	3D assemblies	mostly nanoparticle assemblies	medium	low	low
GLAD	tens of nm to µm	3D helices	metals, oxides, semiconductors	medium	medium to high	low
EBL/FIB	sub‐10 to tens of nm	2D/quasi‐3D	broad	very high	low	low
NIL	tens of nm	2D/quasi‐3D	resist‐compatible	high	high	low
TPL/LDW	sub‐µm to µm	3D	photosensitive materials	high	medium	medium

### 1.2 Challenges of Light‐Induced Chirality

The descriptor‐based comparison shows that different fabrication strategies are suited to different engineering requirements (Table 1). Chemical synthesis, self‐assembly, GLAD, and lithography remain indispensable in their respective application windows. Light‐directed strategies are not universal replacements, but they are particularly valuable when non‐contact processing, direct handedness selection, 3D structuring, dynamic reconfiguration, or site‐selective near‐field chemistry is required. Despite the above‐listed advantages, light‐directed chiral fabrication also faces several challenges. The first is material limitations. Most systems require photosensitive components, which limits to materials with suitable optical properties. This situation has been changing with emerging processability of metals and semiconductors using spiral vector beams or CPL [48, 52, 53]. The technical challenges mainly arise from the high throughput and uniformity for large‐scale manufacturing and integration with conventional nanofabrication. Last but not least, fundamental understanding of predictive models on how light transfers its chirality to matter is ambiguous and a guideline for fabrication parameter selection is missing. These features and issues motivate the focus of this review.

### 1.3 Scope of this Review

Recent developments in light‐induced chiral fabrication, particularly using CPL combined with LDW, have begun to bridge this gap. These methods offer a unique combination of spatial control, scalability, and the ability to directly pattern chiral structures without complex assembly steps. This review provides a comprehensive analysis of current fabrication strategies, with particular emphasis on understanding why light‐induced approaches are emerging as the most promising route for practical applications. Unlike previous reviews that typically focused on chirality transfer in plasmonics [54], or CPL‐facilitated growth [55] and applications (e.g., biosensing and biomedical applications) [12, 24] or particular chiral materials and chiral structures [13, 18, 56], we present a unified framework encompassing all major light‐based approaches for imprinted chirality in nanomaterials, such as LDW, optical manipulation, CPL‐induced processes, and vortex beam methods. This topic has not been fully covered in previous reviews as many of the advancements have just emerged in the last three years, which makes this review timely and important. Specifically, we will make comparative analysis of different optical methods and provide researchers with clear guidance for technique selection. We also introduce light‐modulated chirality beyond static structures, followed by practical applications enabled by light‐induced chirality. At last, we conclude this review with critical assessment of current limitations and future prospects.

This review is intended for researchers working at the interface of chiral nanophotonics, optical micro/nanofabrication, and device‐oriented nanomaterials. For this audience, the central question is not only how chirality can be generated, but also whether the resulting structures satisfy the engineering requirements of chiral light control, including spectral operation range, feature size, structural dimensionality, handedness selectivity, chiroptical figure of merit, array addressability, material compatibility, scalability, and device integration. We therefore discuss light‐induced chirality not as a universal replacement for chemical synthesis, self‐assembly, or lithography, but as a complementary strategy whose advantages become decisive when direct optical handedness selection, non‐contact writing, dynamic reconfiguration, true three‐dimensional structuring, or site‐selective near‐field chemistry is required.

## 2 Light‐Induced Chirality

Although all the optical‐based approaches toward chiral nanostructures involve the light‐matter interactions, their working mechanisms can be totally different. In this section, we will introduce each optical method with specific examples and their latest progresses. At last, we make a comparative analysis of their advantages and disadvantages for the selection guideline of the fabrication.

### 2.1 Light‐Induced Chirality via LDW

**FIGURE 2:**
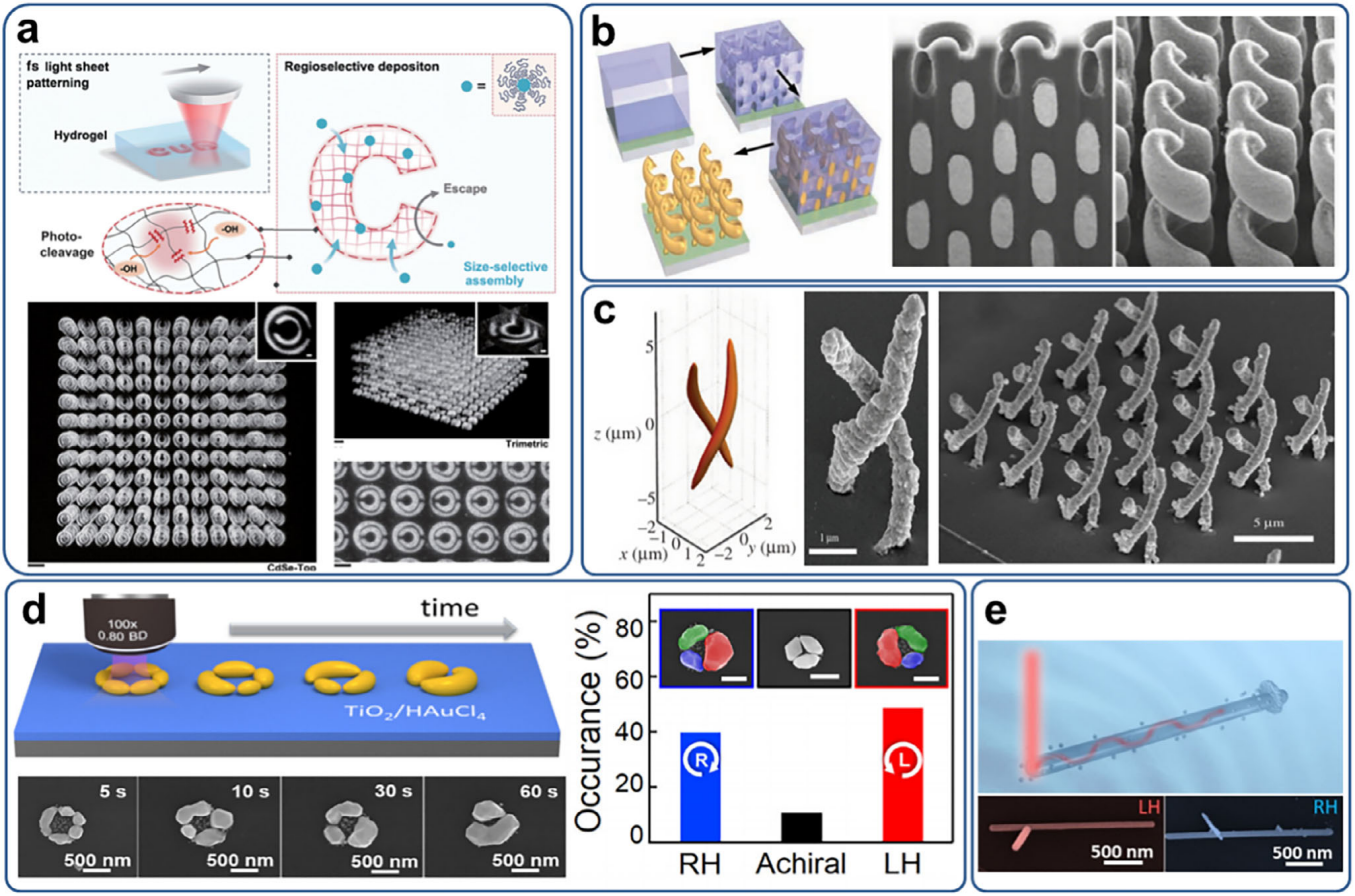
Light‐induced chirality via LDW. (a) Kinetic‐controlled fs‐LDW in doped hydrogels. Top: Schematic illustration of the patterning process, in which complex patterns can be defined with a single exposure and its mechanism of regioselective deposition of materials in a shrunk gel through size selective assembly. Bottom: Top view and trimetric view of a five‐layer split ring resonator (SRR) structure and the SEM image of the top layer after shrinking and dehydration. Insets are SRR unit and slice view. Reproduced with permission [59]. Copyright 2022, American Association for the Advancement of Science. (b) Fabrication of plasmonic nanohelices arrays via LDW and electroplating. Left: schematic of fabrication of plasmonic nanohelices arrays based on TPL and electroplating; Middle: Cross‐sectional SEM of Au‐electroplated structure; Right: SEM of freestanding LH helix array. Reproduced with permission [60]. Copyright 2009, American Association for the Advancement of Science. (c) DLW of Ag double‐helix arrays. Left: Schematic of double‐helix focal field (superposed Laguerre‐Gauss modes); Middle: SEM image of Ag double helix (linewidth ≈180 nm; fabricated with 1030 nm, 240‐fs laser); Right: SEM image of Ag double‐helix arrays.Reproduced with permission [64]. Copyright 2019, Nanophotonics. (d) Laser‐triggered solid‐state growth of chiral ensembles. Left: Schematic of laser‐driven AuNP growth in TiO_2_ (top) and its structural evolution with corresponding SEM images below; Right: Statistical yield of chiral trimers. Reproduced with permission [67]. Copyright 2022, John Wiley and Sons. (e) Laser‐induced branching of Ag NWs. Top: Schematic of plasmon‐directed generation of branched Ag NW; Bottom: SEM images of LH and RH branched Ag NWs with false colors. Reproduced with permission [68]. Copyright 2021, American Chemical Society.

LDW‐based on TPL is well‐known for creating arbitrary two‐dimensional (2D) and 3D structures for functional microdevices [57], which naturally makes it a prominent tool for the fabrication of chiral microobject. However, this method is limited to polymers and photoresists that have large two‐photon absorption cross‐section, which significantly limits their functionality. Although doping/evaporating functional inorganics in/on the helical polymer objects is possible [47, 58], their filling ratio and uniformity cannot be high, which strongly deteriorates their performance as compared to a bulk crystalline piece. This problem hasn't been resolved until recently Chen and coworkers utilized simultaneous shrinkage and assembly process of inorganic NPs in hydrogels to form densely packed 3D structures, which results in an exterior surface roughness smaller than 5 nm and a material filling ratio of ∼60% by volume (Figure 2a) [59]. This strategy has significantly extended the material library for 3D chiral metastructures with diverse functionality.

Alternative approach to generate 3D plasmonic helices is to use positive‐tone resist to build 3D helical microscopic array via TPL and then do electroplating of Au (Figure 2b left panel) [60]. As a result, a gold‐helix photonic metamaterial is generated after removing the polymer template (Figure 2b right panel), which shows broadband circular polarization response as it combines resonant coupling of helical and Bragg modes, offering a compact alternative to conventional quarter‐wave plates. To increase the transmission of the chiral metamaterials, Duan and coworkers further compressed the 3D helical Au into 2D chiral Au metasurfaces, which is also fabricated via TPL but followed by vapor deposition. Such 2D chiral metasurfaces retain broadband optical activity and high transmission with polarization conversion up to 97% [61].

Although these post‐treatment strategies either via electroplating or evaporation can bring new functionality to the inert photoresist, they add to the complication of the operation, error and cost [62]. Direct generation of well‐defined metallic and semiconductor nanostructure with LDW is more attractive and challenging. Early attempt to generate Ag nanostructures with fs‐laser seems possible but their surface roughness and structural integrity appear poor [63], which makes it challenging for 3D chiral metallic objects. Direct generation of double helical arrays via engineering the beam wavefront is more efficient, which can be easily tunable with spatial light modulator (Figure 2c) [64]. Such Ag double‐helix array exhibits pronounced optical chirality in a wide wavelength range from 3.5 to 8.5 µm.

The main challenge of direct fabrication of metallic nanostructures via laser writing is their controllability on the size and surface roughness is poor as they are essentially based on a colloidal process of photochemistry, which involves lots of perturbation such as Brownian motion, reaction kinetics, etc. Bringing photochemistry in solid state can eliminate most of the side effects of solution‐based processing, making it ideal for on‐chip integration [65, 66]. With photochemical reduction of Au ions in TiO_2_ matrix and slow inward‐diffusion‐feeding based on concentration gradient, faceted crystalline Au NPs are formed in a ring‐shaped assembly on the surface [67]. With continued irradiation, strong photothermal effect further melts and merges the Au NPs to form tetramers, trimers and dimers with prolonged irradiation time (Figure 2d left panel). Especially for the trimers, equal occurrence of left‐handed (LH) and right‐handed (RH) enantiomers can be visualized, which show prominent chiroptic response (Figure 2d right panel) [67]. But this chiral appearance is very random, which can be improved with assistance with light manipulation. Wang, et al. demonstrated irradiation location‐controlled branching of Ag NWs which shows enantio‐selectivity [68]. This is realized by controlling the irradiation location relative to the NWs either left or right, which generates Ag NPs in the proximity of the laser focal point (Figure 2e upper panel). The field gradient of surface plasmons and the photothermophoresis further attract and push the generated Ag NPs to the other end of Ag NW, which accumulate into Ag flakes attached to either the left or the right side of the Ag NW, forming branched enantiomers (Figure 2e bottom panel). This process combines the nucleation, manipulation, assembly, and growth in one step, which greatly simplifies conventional LDW process. However, its accuracy and reproducibility is greatly compromised as both the uniformity of the Ag seeds and the growth process is hard to control in aqueous environment. Therefore, direct light manipulation of uniform colloidal building blocks into chiral assemblies has emerged as another facile and tunable strategy for light‐directed chirality [69, 70], which will be further discussed in the next section.

### 2.2 Light‐Induced Chirality via Laser Manipulation

**FIGURE 3:**
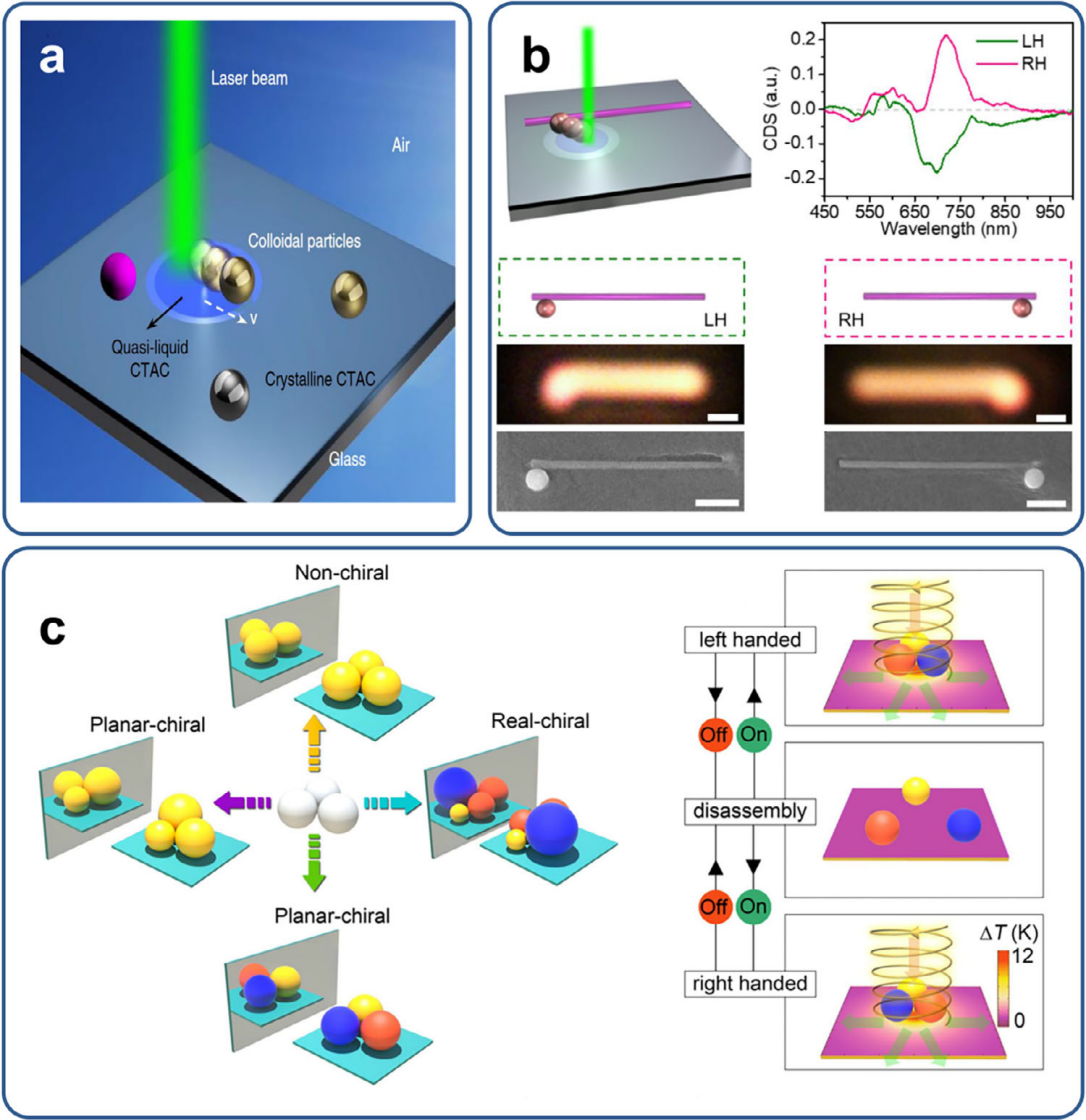
Light‐induced chirality via laser manipulation. (a) Schematic of CTAC surfactant‐mediated OPN manipulation on solid substrates. Reproduced with permission [77]. Copyright 2019, Springer Nature. (b) Schematic of LH silicon nanoparticle‐nanowire chiral structure; CDS spectra showing mirror‐symmetric responses for LH and RH configurations, with corresponding optical and SEM images. Reproduced with permission [78]. Copyright 2021, American Chemical Society. (c) Left: Chirality origin in colloidal meta‐molecules. The balls with different colors represent colloids with different materials. Right: Working principle of the reconfigurable opto‐thermophoretic assembly of chiral meta‐molecules. The red and green arrows represent the incident and scattering light, respectively. Reproduced with permission [79]. Copyright 2019, Elsevier.

Laser manipulation via optical forces provides a more facile and versatile way toward complex colloidal assemblies including chiral nanostructures with reconfigurability [71]. However, due to the diffraction limit of light and strong perturbation of Brownian forces from liquid environment, its operation precision and stability of the nanoassemblies are poor with conventional optical manipulation [72]. Direct laser manipulation on solid substrate can avoid the interruption of liquid environment [73], but it is mostly restricted to 1D movement with opto‐thermo‐mechanical effect [74, 75] or surface acoustic waves [76], which are inefficient for making 3D chiral nanostructures. A recent breakthrough of arbitrary optical manipulation on solid substrate developed by Zheng's research group is based on optothermally‐gated photon nudging (OPN), which operates in a solid‐phase environment [77]. In OPN, a thin surfactant layer introduced between NPs and the substrate acts as an optothermal gate. Under laser illumination, optical heating induces a local phase transition in the surfactant layer, eliminating van der Waals friction and enabling non‐contact manipulation of NPs via optical scattering forces (Figure 3a) [77]. This technique allows for dynamic patterning of nanostructures with nanoscale accuracy, while facilitating in situ optical characterization through dark‐field spectroscopy.

The immediate implication of this strategy is that we can use light to control the formation of chiral assembly in a reconfigurable manner [78]. For instance, silicon nanoparticles (SiNPs) and silicon nanowires (SiNWs) can be assembled into chiral nanostructures by optically nudging the SiNP along the SiNW, transitioning between LH, achiral, and RH configurations (Figure 3b) [78]. This dynamic manipulation directly tailors the chiroptic response as demonstrated by the distinct circular differential scattering (CDS) spectra of each configuration. The observed chiroptic response originates primarily from the resonant coupling between the magnetic dipole mode of the SiNW and the magnetic quadrupole resonance of the SiNP, creating a direct link between structural geometry and optical chirality. The ability to control and reassemble these structures on demand significantly enhances the versatility of chiral metamaterials, making them ideal for applications in nanophotonics and biosensing [78].

Further advancements have been made in the all‐optical assembly of chiral meta‐molecules, where colloidal particles themselves act as “meta‐atoms” [79]. This strategy employs opto‐thermoelectric fields to achieve atom‐by‐atom manipulation and reorganization into chiral configurations, allowing for in situ measurement of their chiroptic response. The combination of light‐generated thermoelectric fields and micelle‐mediated colloidal bonding enables efficient and precise assembly. This approach is versatile and can operate across various materials and particle types, facilitating the creation of chiral meta‐molecules with diverse functionalities (Figure 3c) [79].

It is clear that optical manipulation strategies excel in non‐contact, high‐precision, positioning and dynamic reconfigurability, allowing real‐time manipulation of NPs with minimal interference, thereby preserving material integrity [80]. However, they are typically performed in a serial manipulation fashion, limiting their throughput for large‐scale production of chiral materials [79]. Additionally, the reliance on optical heating raises concerns about thermal management, especially when working with heat‐sensitive materials [81]. The need for carefully controlled environmental conditions, such as specific surfactant layers or electrolyte environments, also restricts the method's applicability across different substrates and materials. As these techniques continue to evolve, enhancing light‐matter interactions by exploiting the contrast between propagating and evanescent fields, for instance through localized surface plasmon resonances, may address these limitations and advance the fabrication of chiral nanostructures with high spatial precision [82].

### 2.3 Light‐Induced Chirality With the Assistance of Surface Plasmons

Surface plasmons, which are the collective oscillations of free electrons at the interface between metal and dielectric materials, have become a fundamental concept in nanophotonics [83]. Their ability to significantly enhance light‐matter interactions is key to their wide range of applications. When light excites the free electrons in metal, these oscillations strengthen the local electromagnetic fields at the metal's surface, leading to remarkable optical effects [84]. In the context of chiral nanostructure fabrication, surface plasmons offer two major advantages, which are the enhancement of local fields and the generation of asymmetric optical forces [85]. The presence of plasmonic nanoseeds amplifies these local fields, improving the interaction between light and matter, which in turn aids in the controlled assembly of chiral structures [86]. On the other hand, even in the absence of plasmonic nanoseeds, surface plasmons can still induce chirality by creating asymmetric optical forces, allowing chirality to be controlled through light manipulation [87]. These unique properties make surface plasmons an extremely versatile tool for creating chiral nanostructures, either by amplifying interactions with plasmonic nanoseeds or by leveraging purely optical methods. This unique feature sets the stage for a deeper exploration of how surface plasmons contribute to chirality under different conditions as we will examine in the following sections.

#### 2.3.1 Fundamental Principles

**FIGURE 4:**
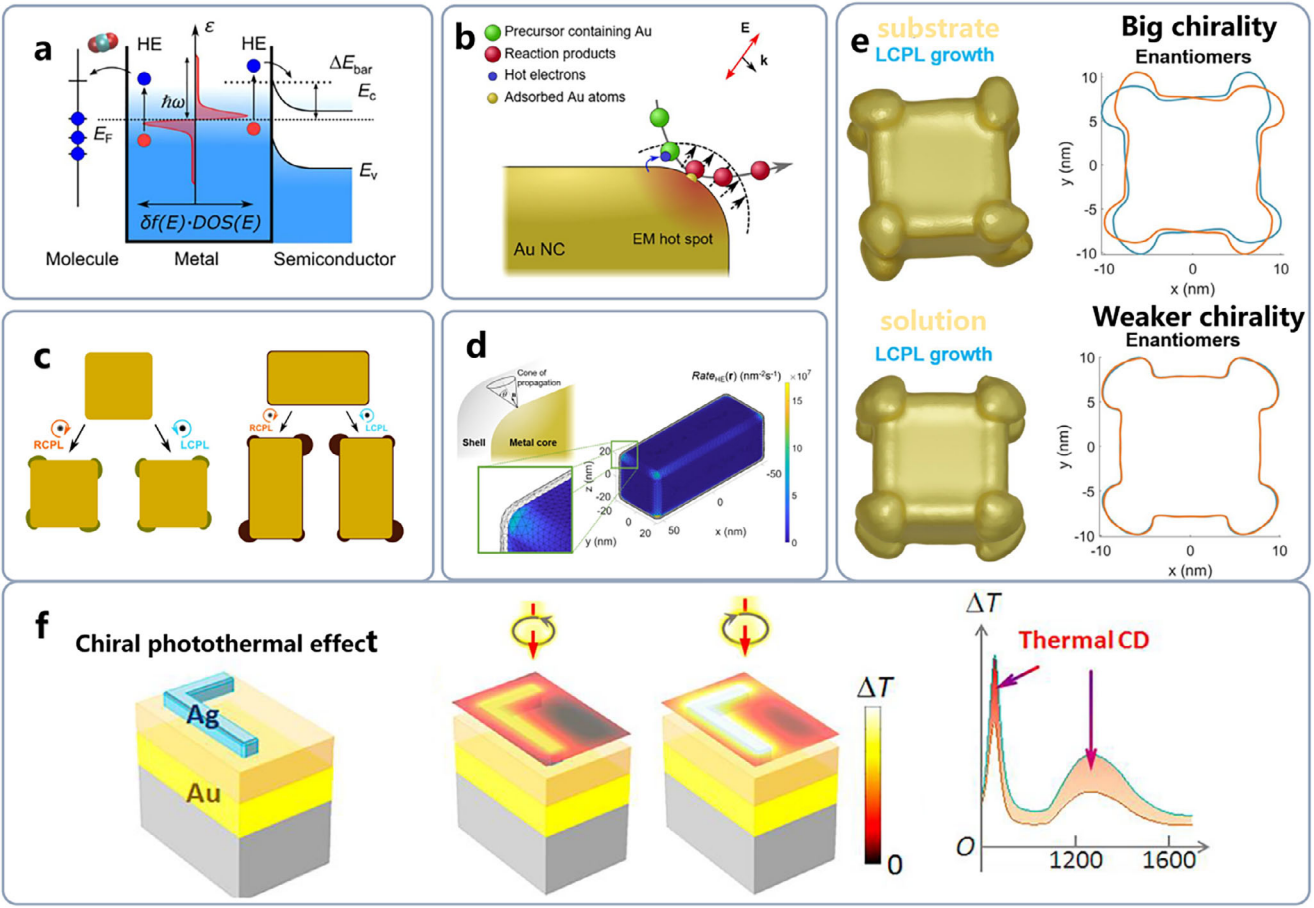
Fundamental principles of light‐induced chirality via CPL. (a) Schematic diagram depicting hot electron injection from the plasmonic metal to its environment. The electrons need sufficient energy to traverse an interfacial potential barrier or reach empty molecular orbitals. A typical energy distribution of excited carriers in a plasmonic NC (red curve) is overlaid over the metal. (b) Diagram depicting the general mechanism of ion aggregation capable of leading to NC growth. (c) Schematic of chiral evolution of an Au nanocube or nanobar controlled by LH and RH CPL. (d) Model of the Au@PbO_2_ system and the Rate_HE_(r) profile at the interface of the two materials under λ = 550 nm illumination. The outer mesh corresponds to the outer surface of the PbO_2_ layer. The inset diagram depicts the cone with an opening angle of 2θ = 120° that propagates from each point at the Au NC's surface. (e) Mesh geometries of the cube after 5 steps of growth under LCPL for planar (top panel) and colloidal (bottom panel) conditions. Right panels show a cross section of both geometries at a z plane 7 nm above their centers. Reproduced with permission [88]. Copyright 2021, American Chemical Society. (f) Photothermal‐induced g‐factor (g_T_) in L‐shaped chiral metamaterial absorbers. Left: schematic of the geometric model; Middle: temperature rise profiles of L‐shaped metamaterials under LCP and RCP illumination; Right: photothermal CD spectra of the L‐shaped metamaterials. Reproduced with permission [94]. Copyright 2018, American Chemical Society.

In plasmon‐assisted light‐induced chirality, nonequilibrium hot high‐energy carriers act at the microscopic level that turns an achiral seed into a chiral product [88]. Alternatively, local photothermal effects can create chiral patterns that may be either permanent or temporary [89]. Figure 4 illustrates both mechanisms, and the equations below provide an overview of the corresponding formalisms.

Surface plasmons compress the electromagnetic field at the metal‐dielectric interface, and the confined near fields promote nonequilibrium hot electrons that overcome interfacial barriers and initiate surface chemistry [90, 91, 92]. As shown in Figure 4a, the hot‐electron excitation/injection rate Rate_HE_(**r**) defined on the seed surface serves as a site‐selective predictor for where material is added or removed in the first step. The helicity contrast is encoded by the differential rate map

(1) $$\Delta \mathrm{Rat}e_{\mathrm{HE}}\left(r\right)=\mathrm{Rat}e_{\mathrm{HE},\mathrm{LCP}}\;\left(r\right)-\mathrm{Rat}e_{\mathrm{HE},\mathrm{RCP}}\left(r\right)\;$$

Opposite optical helicity writes mirror‐related hot‐carrier patterns on the same achiral seed (Figure 4b). As corners, edges, and gap‐like features host plasmonic hotspots with large ΔRate_HE_, continued exposure pushes the geometry away from mirror symmetry, which results in the enantiomorph selected by the CPL helicity (Figure 4c).

Such a differential rate map can only predict where the chiral growth may initiate, but cannot fully determine the final chiral morphology as the overall growth is a nonlinear iterative process where the optical response evolves with the geometry. For growth mapping, the interfacial hot‐carrier response is projected into the outer dielectric overlayer through a cone with opening angle 2θ = 120°, and only voxels inside the cone are allowed to grow with an overlayer refractive index of 2.3. Iterating a cycle of rate mapping, growth mapping, mesh updating and optical recomputation yields progressive increase in the CD and g‐factor spectra, which can be used to track the initial ΔRate_HE_ of the seed (Figure 4d) [88].

The illumination geometry is crucial in determining the potential magnitude of such chiral growth. When the system must jump from zero to finite chirality at the outset, a planar illumination preserves the fixed phase relation between the incident wave and the seed, producing a stronger and cleaner ΔRate_HE_ than colloidal illumination. After five growth iterations, the CD and g‐factor under planar conditions exceed those under colloidal conditions by more than an order of magnitude. The two CPL enantiomorphs separate clearly in shape under planar illumination, whereas their cross‐sections nearly overlap in colloidal conditions (Figure 4e). This contrast originates from the direction‐fixed ΔRate_HE_ of the seeds established in the first step [88].

Viewed through energy flow, the same near‐field hotspots that feed Rate_HE_ also feed the electromagnetic absorption (*Q_h_*). With fixed geometry, this absorption drives transient chirality imprinting. In the metasurface picture, the absorbed power density is

(2) $$Q_{h}\left(r,t\right)\;=<\;j\left(r,t\right)\cdot E\left(r,t\right)\;{>}_{t}=Q_{h}\;\left(r\right)F_{\mathrm{pulse}}\left(t\right)$$

with a Gaussian pump envelope *F*_pulse_(*t*), which is typically 100 fs in simulations, defining the pump‐probe timeline. The ensuing thermal dynamics follows a lattice‐only one‐temperature (1T) model or the coupled two‐temperature (2T) model:

(3) $$C_{e}\left(T_{e}\right)\partial_{t}\;T_{e}=\nabla \cdot \left(k_{e}\left(T_{e}\right)\nabla T_{e}\right)-G\left(T_{e}-T_{l}\right)+Q_{h}\left(r,t\right)\;$$

(4) $$C_{l}\left(T_{l}\right)\partial_{t}\;T_{l}=\nabla \cdot \left(k_{l}\left(T_{l}\right)\nabla T_{l}\right)+G\left(T_{e}-T_{l}\right)\;$$

In this model, the coupled fields *T_e_* (***r***,*t*) and *T_l_* (***r***,*t*)are the electron (e) and lattice (l) temperatures. *T*_*e* _is defined only in the gold, whereas *T_l_* is defined everywhere (as in the 1T model), so electronic energy relaxes into phonons until equilibrium. The balance equations *C*_e_(*T*_e_)∂_t_ *T*_e_ =  ∇ · (*k*_e_(*T*_e_)∇*T*_e_) − *G*(*T*_e_ − *T*_l_) + *Q*_h_(**r**,*t*) and *C*_l_(*T*_l_)∂_t_ *T*_l_ =  ∇ · (*k*_l_(*T*_l_)∇*T*_l_) + *G*(*T*_e_ − *T*_l_) use the volumetric heat capacity *C*(*T*) and the (temperature‐dependent) thermal conductivity*k*(*T*). In metals, the total conductivity is written as *k*(*T*) ≈ a*k*_e_(*T*_e_) + b*k*_l_(*T*_l_) with a = 0.99 and b = 0.01 for gold. The electronic heat capacity is defined with the Debye approximation,*C*_e_(*T*_e_) ≈ 71.4*T*_e_ J · m^−3^ · K^−1^, while *C_l_* (*T_l_*) and *k*_l_(*T*_l_) are taken from COMSOL libraries. The G term is the electron–phonon coupling constant with a value of 2.78 × 10^16^ W · m^−3^ · K^−1^ [93].

Indeed, upon illumination with chiral light, planar L‐shaped absorbers are able to generate a prominent asymmetry in their local temperatures, which yields a photothermal g‐factor (g_T_) equivalent to their the optical g‐factor (g_CD_) with magnitude up to 0.5 (Figure 4f) [94]. This chiral metamaterial heater can be potentially used for surface photochemistry and to create bolometers sensitive to the circular polarization of incident light.

The temperature feeds back into the metal permittivity, which also leads to the shift and broadening of surface plasmons while the geometry remains unchanged. The Drude form baseline is

(5) $$\varepsilon_{\mathrm{metal}}^{\mathrm{bulk}}\;\left(\omega \right)=\varepsilon_{b,\mathrm{Drude}}\;-\frac{\omega_{p0}^{2}}{\omega \left[\omega +i\gamma_{D0}\right]}\;$$

and the explicit temperature dependence reads

(6) $$\varepsilon_{\mathrm{metal}}^{\mathrm{bulk}}\;\left(\omega ,T\right)=\varepsilon_{b,\mathrm{Drude}}\;\left(T\right)-\frac{\omega_{p}^{2}\left(T\right)}{\omega \left[\omega +i\gamma_{D}\left(T\right)\right]}\;$$

where ω_P_(T) varies with carrier density and volumetric thermal expansion α_v_ ≈ 3α_L_, and

(7) $$\gamma_{D}\left(T\right)=\gamma_{D0}+\beta \left(T_{l}-T_{0}\right)c_{e-\mathrm{ph}}$$

captures additional electron–phonon damping. Substituting the space‐time temperature *T*(**r**, *t*)back into ε(ω, *T*) yields the CPL‐conditioned time‐dependent permittivity ε(**r**, t; CPL). A field‐level CD can be obtained

(8) $$CD_{\left|E/E_{0}\right|}={\left|\frac{E}{E_{0}}\right|}_{\mathrm{LCP}}^{2}-{\left|\frac{E}{E_{0}}\right|}_{\mathrm{RCP}}^{2}\;$$

which traces how transient heating writes helicity into the optical response of achiral or chiral metastructures over picosecond to hundred‐picosecond windows [89].

#### 2.3.2 Light‐Induced Chirality With the Presence of Plasmonic Nanoseeds

**FIGURE 5:**
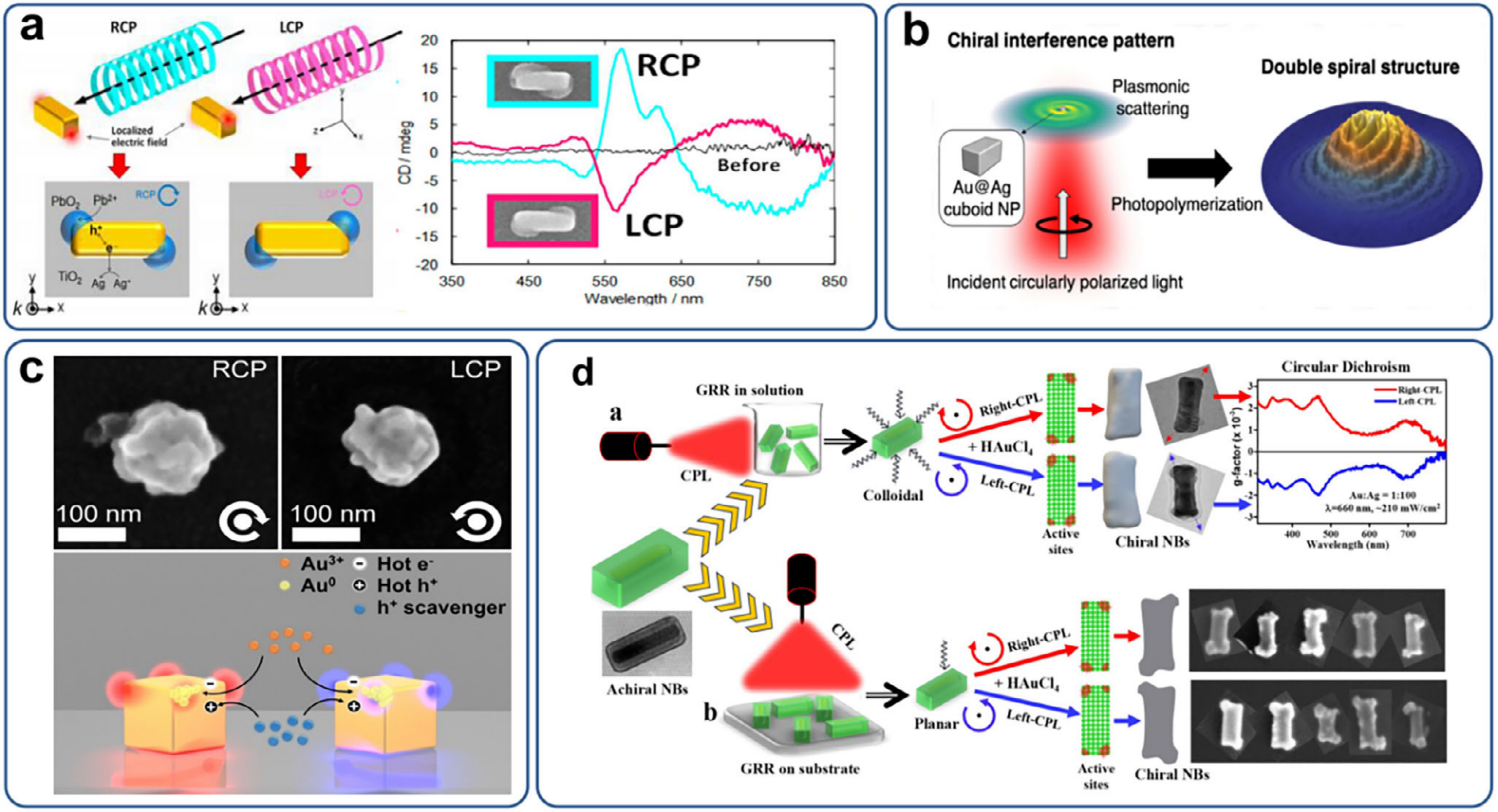
Light‐induced chirality enabled by surface plasmons. (a) Plasmon‐induced charge separation. Left: Schematic of chiral nanostructure fabrication using CPL as the chiral source; Right: CD spectra of chiral hybrid PbO_2_/Au nanostructrues. Insets are the corresponding SEM images.Reproduced with permission [50]. Copyright 2018, American Chemical Society. (b) Schematic of plasmon‐CPL interference patterns with Au@Ag cuboids transferred to polymeric surface reliefs via photopolymerization. Reproduced with permission [100]. Copyright 2024, American Chemical Society. (c) Hot electron‐mediated chiral growth. Top: SEM images of RAuNCs and LAuNCs grown under RCP and LCP irradiation; Bottom: Mechanism of polarization‐dependent hot electron generation for Au^3+^ reduction and chiral growth. Reproduced with permission [101]. Copyright 2024, John Wiley and Sons. (d) Chiral transformation of nanobars. Top: Schematic of galvanic replacement under R‐/L‐CPL with rotational averaging and their CD g‐factor spectra; Bottom: Schematic of directional CPL illumination with surface‐immobilized NBs and their SEM images. Reproduced with permission [102]. Copyright 2024, American Chemical Society.

Moving beyond laser manipulation, plasmon‐assisted growth under CPL writes chirality directly through near‐field‐controlled redox chemistry. Tatsuma's group demonstrated this mechanism by employing CPL as the chiral source to activate Au nanocuboids on a TiO_2_/ITO substrate. The crux of the method lies in the handedness‐dependent localization of twisted electric fields at specific corners, where subsequent plasmon‐induced charge separation (PICS) directs the hot‐hole oxidation and deposition of PbO_2_. This site‐selective modification ultimately yields chiral nanostructures with a clear sign reversal in circular dichroism (CD) across the visible and near‐infrared range. The single hybrid particle can reach an enantiomeric excess of 43% and an ellipticity close to 20 mdeg (Figure 5a) [50]. A practical limitation appears when the dielectric overgrowth blocks the hot spots and weakens direct electromagnetic access. They further addressed this issue with all‐metal architectures [95]. They adopted a Ag^+^/citrate precursor system, where the CPL triggers hot‐electron reduction based on Ag nanoplate arrays. Finally, the Ag nanoplates evolve into chiral plasmonic metasurfaces on a glass support. The exposed hot spots in the resulting nanostructures give rise to strong chiroptic responses, and the fabrication process remains solution‐compatible over large areas. Here, the symmetry does not need to be pre‐encoded in the seeds. Tatsuma and coworkers used isotropic and symmetric Au nanodisks as plasmonic precursors. Under CPL irradiation in a Ag^+^ and citrate solution, the initial Ag deposition on the Au nanodisks start at an arbitrary site, breaking the symmetry and giving rise to an anisotropic and asymmetric electric field distribution. The asymmetry, attributed to the interference between the dipole LSPR mode and a higher‐order resonance such as a hexapole, leads to chiral shaping of Au‐Ag nanostructures and chiroptic responses. The electric‐field distribution is inverted to the mirror image when the handedness of CPL is switched and continued illumination directs handed growth and fixes the handed state [96].

Spectral control adds programmability to the seed‐assisted chiral plasmonic growth. Tang's group used Au bipyramids on TiO_2_/ITO as the seeds and showed that the deposition of PbO_2_ is wavelength‐dependent. Illumination with 488 and 532 nm light concentrates the growth of PbO_2_ near the waist, whereas 660 nm light irradiation confines the growth at the tips. These observations show that wavelength‐dependent plasmonic field distributions are more important than surface lightning rod effects in localizing the photodeposition. The role of AgNO_3_ is to consume electrons, thereby increasing the lifetime of photogenerated hot holes and promoting the oxidation of Pb^2+^. Despite the larger extinction coefficient at longer wavelengths, photodeposition with 660 nm illumination proceeded much slower, which is consistent with the decreased oxidative strength of hot holes generated via intraband transitions [97]. The same wavelength‐site correlation holds for different shapes. In a follow‐up study, they further positioned colloidal Au nanoprisms on TiO_2_/ITO substrate and observed side deposition with 532 nm light illumination with higher dissymmetry g‐factor of ∼3.6×10^−3^, and tip‐biased deposition at 660 nm with a more symmetric response. Field simulations and experimental spectra are in good agreement, corroborating the wavelength‐dependent localization of photodeposition. Moreover, precursor crystallinity and uniformity emerge as decisive factors for achieving high ensemble g‐factors and clean CD lineshapes, as inhomogeneous deposition and substrate adhesion can lower the overall dissymmetry [98]. Surface chemistry supplies a second control factor. In CTAB‐capped Ag nanorods derived from Au‐bipyramid seeds, they found halide‐rich micelles bind lateral (100) facets more strongly than tip (111) facets, suppressing sidewall reduction and preserving tip activity. As a result, under CPL, early growth becomes tip‐biased and evolves into hook‐like profiles and the reinforced near‐field dissymmetry drives large optical responses with ensemble g‐factors approaching about 0.05 near 800 nm. Removing or altering ligands diminishes spatial selectivity and weakens CD, underscoring the coupling between field distribution and facet‐selective passivation [99].

Beyond near‐field hotspot writing, the interference between the plasmon scattered field and the incident CPL produces a double spiral optical pattern around achiral NPs that can be developed by photopolymerizing into chiral surface relief structures [100]. The handedness and periodicity of the spiral depend on the incident wavelength and nanoparticle geometry through the hybridization of transverse and longitudinal plasmon modes and their phase relationship, enabling wavelength‐tunable structural patterning (Figure 5b) [100].

Mechanistic evidence at the single particle level closes the loop. CPL induces a mirror‐asymmetric distribution of hot electrons on achiral Au nanocubes, and the site‐selective deposition replicates the optical handedness in the chiral morphology. The same principle applies to the chiral growth of Ag or Pd on Au seeds, demonstrating the broad applicability of this chirality‐transfer strategy (Figure 5c) [101].

In colloidal environments, symmetry breaking can survive orientation averaging. Randomly rotating Au@Ag nanorods undergo plasmon‐assisted galvanic replacement under CPL can evolve into chiral shapes. Immobilizing the particles on substrate or using directional illumination amplifies the asymmetry, and the observed selectivity aligns with hot electron‐assisted electrochemistry rather than simple photothermal heating under the reported fluxes (Figure 5d) [102].

These recent developments define the strengths of plasmonic seed‐assisted routes, which is capable of generating ultrafine chiral features in the scale far below subwavelength. However, it also poses several critical constraints, such as extremely low efficiency and scalability that hamper this strategy from practical applications. With these benefits and limits in view, the discussion now turns to seed‐free strategies that dispense with strong plasmonic localization and hot carrier supply but still write chirality using CPL or structured light fields. We will discuss their mechanisms, controllability, and integration prospects as compared to the seed‐assisted paradigm outlined here [103, 104, 105].

#### 2.3.3 Light‐Induced Chirality Without the Presence of Plasmonic Nanoseeds

**FIGURE 6:**
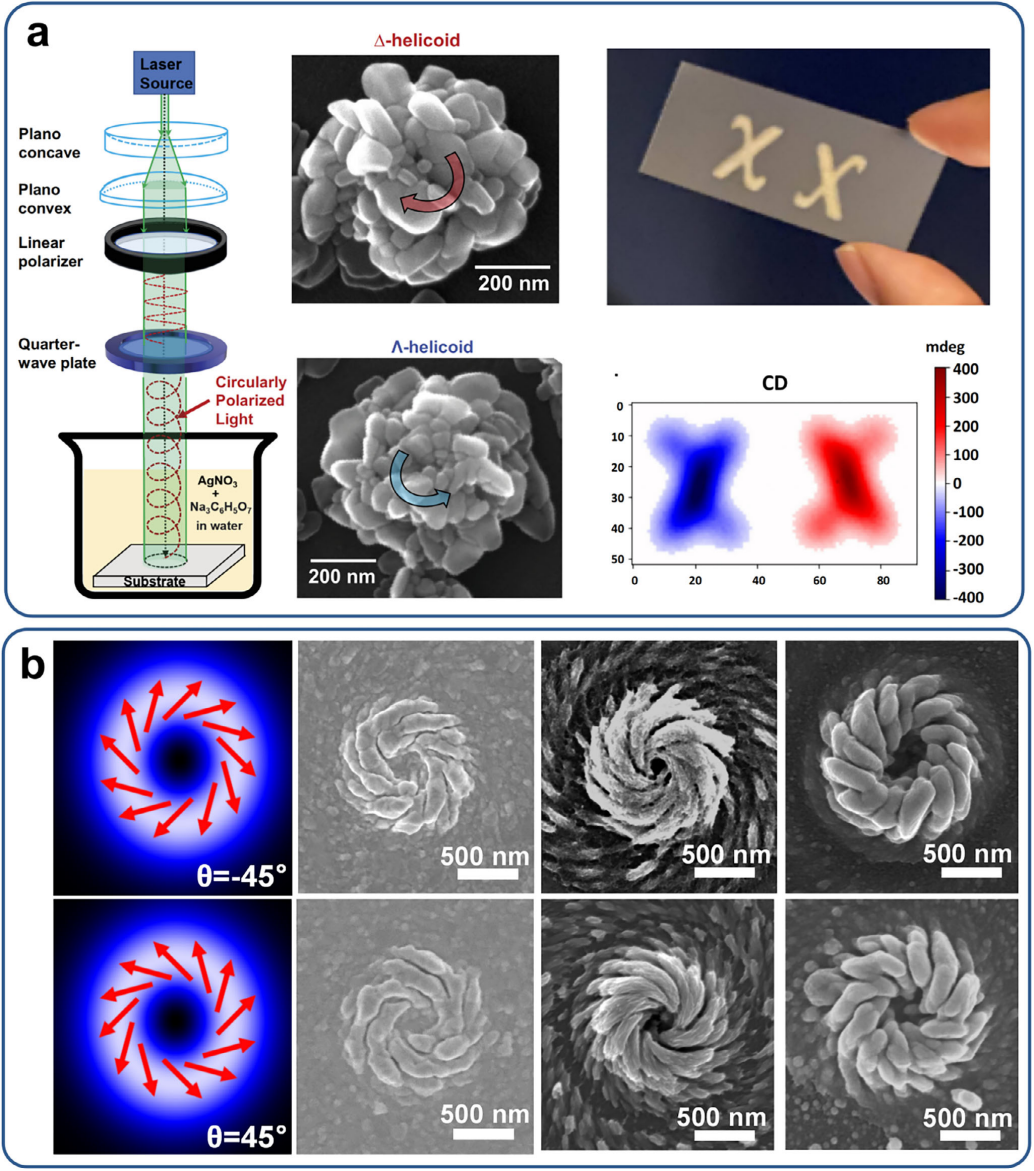
Light‐directed chiral nanostructure fabrication. (a) CPL‐driven silver nanohelicoid synthesis: Left: Schematic of CPL‐printing for substrate‐normal nanohelicoid forests; Middle: SEM images of LH (L‐CPL‐formed, χ) and right‐handed (R‐CPL‐formed, χ) nanohelicoids on ITO/glass; Right: Patterned demonstration with mirrored “χ” letters printed by 532 nm L‐CPL/R‐CPL (top) and corresponding CD mapping via Mueller matrix polarimetry (bottom, spatial resolution: 1 mm). Reproduced with permission [52]. Copyright 2024, PNAS. (b) Top: Simulated beam profile at θ = −45°; Bottom: Simulated beam profile at θ = +45°; Right: Corresponding SEM images of the complex (chiral) patterns of different compositions. Red arrows indicate polarization orientation. Reproduced with permission [48]. Copyright 2023, Springer Nature.

In contrast to seed‐assisted schemes, seed‐free light‐induced chirality can generate arbitrary patterns with relatively larger scalability while preserving geometric programmability. Prior to the polarization‐directed chiral growth, early work focuses on the polarization‐directed anisotropic growth. Linearly polarized light can guide directional deposition or elongation through polarization‐dependent near‐field localization, hot‐carrier generation, photothermal gradients, and anisotropic photochemical reaction pathways [106, 107]. Although such linear growth does not itself generate handedness, it provides the mechanistic foundation for chiral growth when the polarization direction is spatially rotated, temporally varied, or converted into circular, vectorial, or vortex fields. In this sense, polarization‐directed linear growth represents an important precursor to light‐directed chiral morphogenesis [108, 109]. Later, both theoretical analysis [88, 110] and experimental validation [111] have suggested CPL can be directly used for chiral plasmonic nanoparticle synthesis [49] and self‐assembly [112]. But as they are mostly solution‐based, arranging them at desired location for on‐chip integration becomes challenging. More recently, Kotov et al. have shown 3D Ag nanohelices can be directly formed on solid supports with CPL DLW, which yields mirror enantiomers over centimeter‐scale areas on glass, ITO, Si, and elastomer substrates [52]. The resulting arrays show appreciable chiroptic response with large g‐factors, achieved without chiral ligands or post processing (Figure 6a) [52]. Spatially structured vector beams [48] or vectorial holography [113] offer a complementary route. Under continuous wave irradiation, photochemical redox reaction at the focal spot generates a roughened substrate and nanoseeds, which creates a self‐aligned near field that enhances photochemical growth along the polarization direction. By tailoring the polarization distribution into spiral configuration, this directed growth evolves into spiral nanostructures with controllable handedness across metals (Au), oxides (PdO), and semiconductors (CdS), enabling patterned assemblies amenable to device integration (Figure 6b) [48]. For material systems that are polarization‐sensitive, they can form oriented growth along the laser polarization [106]. As such, we can program the laser scanning and polarization to generate twisted micropillar arrays [53].

These optical operations combine chemical cleanliness, ambient processing conditions, and straightforward scalability with programmability through polarization state and wavelength control. Practical limits arise where resonant enhancement is weak or field control is imperfect, since uniformity and throughput are dependent on the accurate polarization/intensity profiles, substrate and precursor reactivity, and cross talk within the arrays [103]. Taken together, seed‐free optical operation demonstrates that spin and spatial polarization structure alone can imprint handed geometry without plasmonic nanoseeds, and this perspective naturally leads to optical vortices that carry orbital angular momentum [114], whose helical phase and annular intensity provide additional levers to sculpt chiral growth and field gradients, which will be discussed in the next section.

### 2.4 Light‐Induced Chirality via Optical Vortex

Optical vortices (OV) encode handedness through a helical wavefront. The OV carries orbital angular momentum (OAM) ***Lħ*** and circular polarization contributes to the spin angular momentum (SAM) ***Sħ***, which together contribute to the total angular momentum ***J* = (*L+S*)*ħ***. Such OAM bears strong optical torques on microobjects, which can be used for rotatory manipulation [115].

**FIGURE 7:**
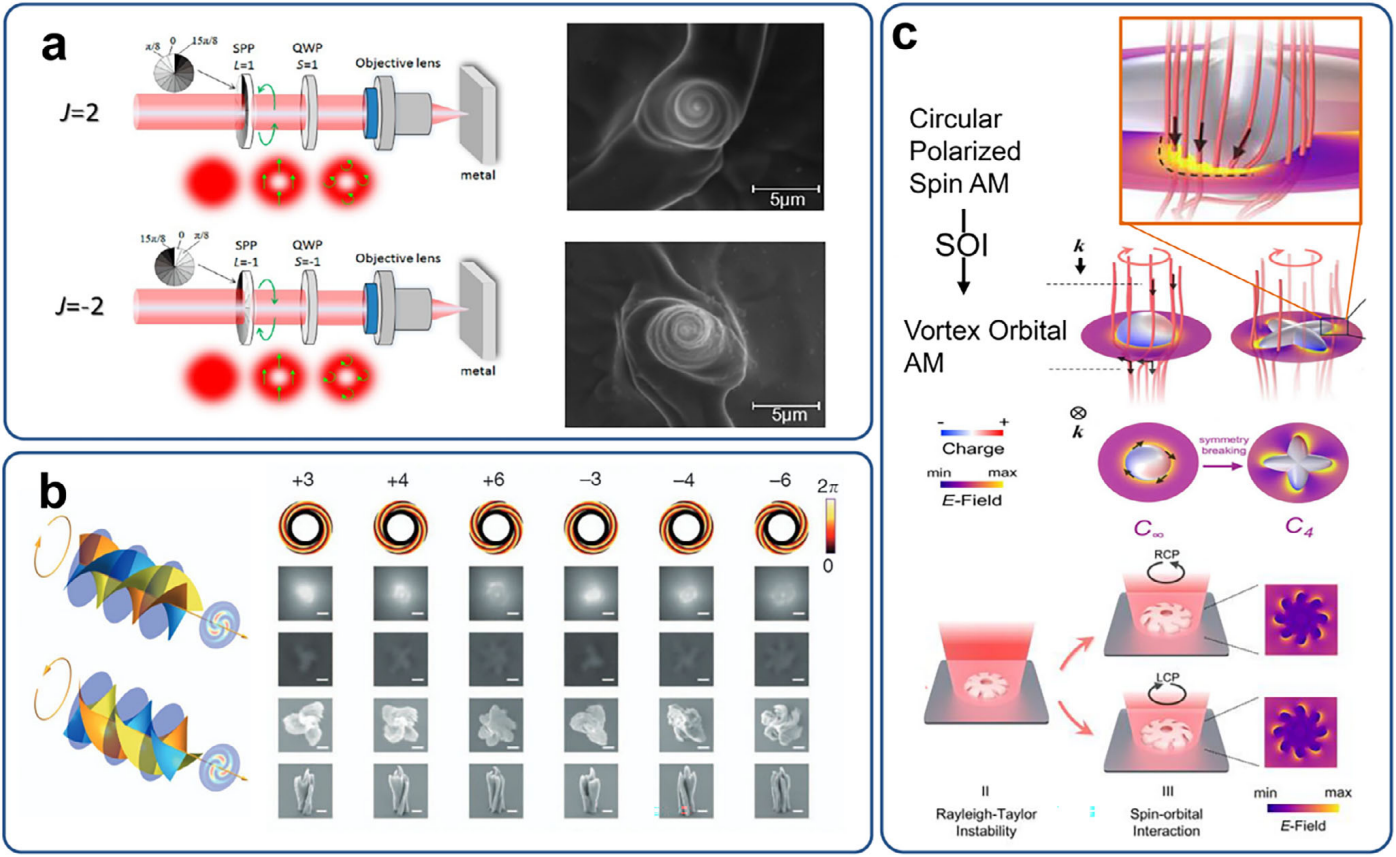
Light‐induced chirality via optical vortex. (a) Optical vortex setup for the fabrication of nanoneedle. Left: Schematic of experimental setup using spiral phase plate (SPP) and quarter‐wave plate (QWP) to generate circularly polarized optical vortex pulses; Right: Top‐view SEM images of fabricated nanoneedle. Reproduced with permission [116]. Copyright 2012, American Chemical Society. (b) Holographic chiral microstructures. Left: Spiral interference pattern from vortex beam‐plane wave interference (*I* = ±3); Right: beam modulated by designed holograms (first row) with different topological charges and the intensity pattern (second row); Optical micrographs of the polymer (third row); SEM images of top‐view (fourth row) and 45° tilted‐view (fifth row) of chiral microstructures. Scale bars are 2 µm. Reproduced with permission [119]. Copyright 2017, Springer Nature. (c) Chiral self‐assembly mechanism. Top: Schematic of spin‐orbit interaction (SOI) around nanosphere under C_∞_ and C_2_ symmetries, showing photon trajectories (red streamlines) and charge distributions; Bottom: CPL‐driven chiral assembly process. Reproduced with permission [121]. Copyright 2025, John Wiley and Sons.

Along with photothermal effect, the pulsed OV beam can drive the azimuthal melt flow toward the intensity null, which the resolidifies the volume into a conical nanoneedle with twisted configuration. Theoretical analysis suggests the gradient forces pull material toward the bright ring, and scattering forces drive it inward. The OAM supplies azimuthal momentum and creates a shear flow around the axis. The twisting direction, either clockwise or anticlockwise, is dependent on the sign of the topological charge of the vortexed beam (Figure 7a) [116]. Specifically, the sign of ***L*** fixes the handedness, and ***S*** = ±1 determines the spiral frequency and texture clarity. Matching signs of ***L*** and ***S*** will tighten the winding while opposite signs will loosen it. The features reach micrometer heights, which terminate in nanosharp tips with minimum curvature about λ/25. A low numerical aperture focus affords a long depth of focus that tolerates minor defocus. If the pulse energy is too high, plasma shielding and roughening suppress the chiral imprint, which defines a relatively tight processing window [117].

In isotropic photoresists, the OV typically produces a nonchiral doughnut voxel. However, when superposing a coaxial plane wave at the focus, a fully three dimensional spiral field can be generated [118]. Based on this principle, Wu et al. utilized a spatial light modulator to display an interfering vortex hologram, enabling the polymerization of 3D chiral microstructures in a single exposure over large areas (Figure 7b) [119]. The number and sense of spiral lobes follow the topological charge, and a reference phase control rotates the pattern in plane, which enables array level registration without point‐by‐point scanning. As the size of the chiral unit generated by OV beam is much larger than the wavelength of light, they exhibit strong OAM chirality characterized by vortical differential scattering, which provides an efficient strategy of chiroptic discrimination of multiscale chiral structures [120].

Although optical vortices can fabricate chiral structures using structured light fields, the optics are often rely on intrinsic beam asymmetry. Another much simpler route based on seed symmetry‐breaking (SSB) effect triggered by femtosecond‐laser‐induced nanoscale Rayleigh‐Taylor instability (nano‐RTI) was recently proposed and realized by Wang, et al. in low‐melting point material system of Sb_2_Se_3_ [121]. Under circularly polarized illumination, a nominally symmetric melt pool loses continuous rotational symmetry. Petal‐like tentacles extend outward due to nano‐RTI, serving as asymmetric seeds. The resulting symmetry breaking, coupled with spin–orbit interaction (SOI), generates a chiral near field that guides the formation of chiral structures without the need for vortex‐phase modulation (Figure 7c).

Overall, OV writing provides deterministic handedness switching by toggling the topological charge, accessing to high aspect ratio metallic features with nanosharp tips, and fabricating three dimensional patterns in isotropic polymers with digitally programmable pitch, lobe number, and in plane orientation in a single shot [118, 122, 123]. Seen in this light, OV complements the plasmon‐assisted route by shifting the primary controls from hotspot geometry to wave front topology and angular momentum [50]. However, as the formation mechanism couples thermal transport, phase change, and fluid dynamics in a material‐dependent manner, its generality and scalability is greatly limited [124].

### 2.5 Comparative Analysis of Light‐Induced Methods

To systematically compare the optical performance of different light‐induced chiral nanostructures, it is necessary to define quantitative metrics that capture their chiroptical responses. Depending on the measurement modality, these responses can be characterized by CD ellipticity, differential absorption, differential scattering, extinction dissymmetry factor, luminescence dissymmetry factor, optical rotation, or helicity‐dependent photothermal response. The physical origin of these responses varies with the system. For instance, plasmonic near fields enhance absorption or scattering asymmetry through local field confinement [50]. Chiral cavities modulate g_lum_ via mode‐selective emission and Purcell effects [125]. Photothermal structures produce helicity‐dependent temperature changes through asymmetric light absorption [126]. Enantioselective optical forces produce piconewton‐scale force differences through circularly polarized light [127]. Establishing such metrics provides a consistent basis for evaluating and comparing the functional performance of diverse light‐induced chiral architectures.

Because the term “chirality enhancement” can refer to different optical observables, we now summarize representative matrix specifically for light‐induced chiral nanomaterials (Table 2). The listed values are order‐of‐magnitude benchmarks which depend on wavelength, excitation power, detection geometry, sample orientation, and ensemble vs. single‐particle averaging. In plasmonic systems, ensemble‐level dissymmetry factors are commonly in the range of 10^−4^–10^−2^ while optimized structures with strong near‐field localization, facet‐selective growth, or ligand‐regulated morphology can reach over 0.1 [25]. In chiral luminescence systems, the luminescence dissymmetry factor is typically smaller, but can approach values on the order of 0.1–0.5 when emitters are placed in strongly chiral photonic hotspots [128]. Photothermal chiral metasurfaces can also display large helicity‐dependent thermal responses, with reported photothermal dissymmetry factors around 0.01 [129]. Enantioselective optical forces acting on single nanoparticles or small arrays under circularly polarized light can be quantified with differences between left‐ and right‐handed enantiomers approaching ∼10 pN [130]. These values provide quantitative context for comparing different light‐induced mechanisms.

**TABLE 2:** Representative metrics and typical ranges used to evaluate chirality enhancement in light‐induced chiral nanomaterials.

Metrics	Definition^a^	Typical range	Reference
CD	*A_L_*‐*A_R_*	mdeg to tens of mdeg	[131]
*g_abs_*	2(*A_L_*‐*A_R_*)/(*A_L_* +*A_R_*)	10^−4^ to10^−2^	[132]
*g_CDS_*	2(*S_L_*‐*S_R_*)/(*S_L_* +*S_R_*)	10^−2^ to10^−1^	[133]
*g_lum_*	2(*I_L_*‐*I_R_*)/(*I_L_* +*I_R_*)	10^−3^ to10^−1^	[132]
*g_T_*	2(Δ*T_L_*‐Δ*T_R_*)/(Δ*T_L_* +Δ*T_R_*)	∼0.01	[129]
Δ*F*	*F_L_*‐*F_R_*	∼10 pN	[130]

^a^ *A*, *S*, *I*, *T*, and *F* represent the absorption, scattering, emission, temperature and optical forces, respectively. L and R represent the parameters obtained under the excitation of LCP and RCP, respectively.

While Table 1 provides a broad engineering‐oriented comparison of representative chiral nanofabrication strategies, Table 3 provides application‐oriented guidance on the method selection of light‐induced chirality. For DLW, it has a higher resolution but limited materials option. For optical manipulation, it can work with different particles but the scalability is poor. Plasmon‐assisted chiral growth can induce high spatial resolution but it requires plasmonic seeds and the scalability is low. Seedless CPL writing can have higher scalability but the number of applicable materials is limited. Vortex beam writing may create complex chiral structures all in one step but its resolution is low. Therefore, different optical methods may be better suited for different applications. For device fabrication, direct writing with CPL is the more likely to be the best method due to its high scalability and low cost [52], while for fundamental research, optical manipulation may work the best as it provides more controllability and material diversity to reveal the material‐structural‐chiroptic correlation [79]. For high‐resolution chiral structure formation, plasmon‐assisted approach may be the most appropriate as it utilizes subwavelength light confinement for near field sculpturing [88]. Vortex or vector beams may be the easiest way to achieve the 3D complex chiral structures [51, 134]. This application‐dependent view provides a more practical framework for selecting light‐induced methods.

**TABLE 3:** Practical Guidance for Selecting Light‐Induced Chirality Methods.

Method	Spatial Resolution	Material Scope	Scalability	Complexity	Application scenarios
DLW	High (∼λ/2)	Photo‐sensitive	Medium	Medium	Polymer‐based chiral arrays
Optical Manipulation	Medium	Broad	Low	High	Models for structure‐property investigation
Plasmon‐Assisted CPL Writing	Very High (<λ/10)	Plasmonic	Low	Medium	Mechanistic study and high‐resolution chiral structures
Seedless CPL Writing	Medium	Limited	High	Low	Large‐area metallic/semiconductor chiral arrays
Vortex Beam Writing	Low	Broad	Medium	High	Complex chiral nanostructures

## 3 Light‐Modulated Chirality

Tunable chiroptic systems, which can dynamically control their interaction with CPL, are valuable for applications ranging from 3D displays and optical telecommunications to pharmaceutical sensing and anti‐counterfeiting technologies [135]. These systems allow real‐time adjustment of optical activity, enabling devices like switchable privacy screens, optical data modulators [136]. In previous section, we show facileness and versatility of light‐induced chirality. Although it can generate enantio‐selective chiral nanostructures in a scalable manner, these structures and their chiroptic properties are mostly fixed once they are formed, which cannot be easily tunable, limiting their functionality. Therefore, light‐modulated chirality is another important aspect of light‐induced chiroptics that has unique advantages of fast speed, enantio‐selectivity, and reversibility as compared to other means such as chemical, mechanical and electric methods [14, 137, 138]. It presents good promise for device integration and all‐optical chiral operation.

**FIGURE 8:**
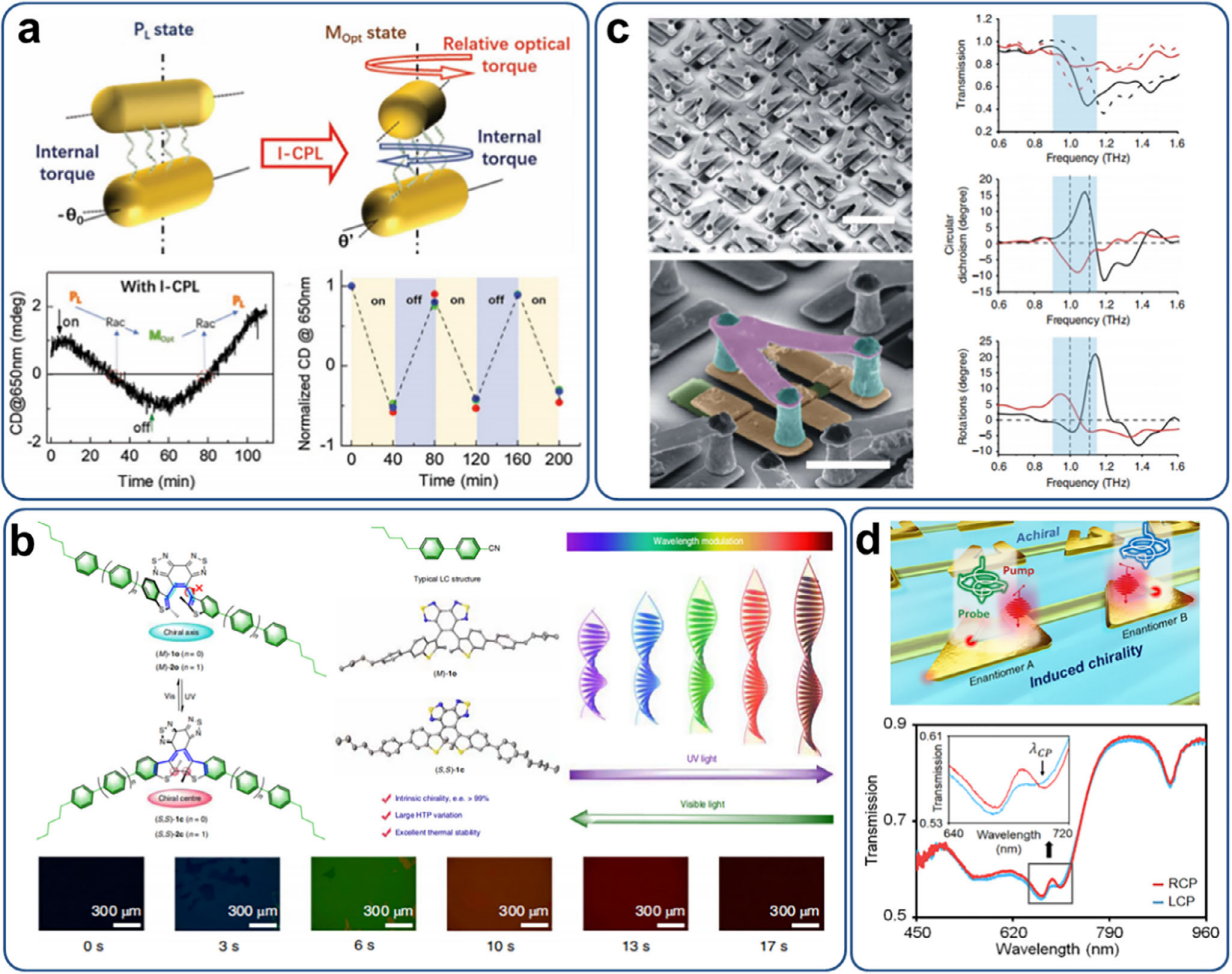
Light‐modulated chirality. (a) Chirality switching in Au nanorod dimers: Schematic of P2→P3 inversion driven by optical (red arrow) and internal torque (blue arrow); CD spectra showing transition states and normalized kinetics (dashed guides: 280 min transition). Reproduced with permission [139]. Copyright 2019, John Wiley and Sons. (b) Schematic of enantioselective chirality transformation; Pitch‐length modulation data showing reflection tuning and reversible cycling under UV–vis irradiation. Reproduced with permission [146]. Copyright 2022, Springer Nature. (c) SEM images of array overview (scale bar: 25 µm; period: 50 µm) and nanostructure detail (scale bar: 50 nm); Polarization spectra showing circular transmission (solid/dashed lines), circulation ellipticity, and ORD transmission with disclination effects (shaded regions). Reproduced with permission [147]. Copyright 2012, Springer Nature. (d) Schematic of polarization‐dependent chiral asymmetry in metasurfaces; Linear optical transmission spectra for left‐ and right‐circularly polarized light with integrated CD region (inset). Reproduced with permission [148]. Copyright 2024, PNAS.

Light‐induced modulation of chiroptic response can be generally classified as light‐induced chiral structure modification and light‐induced electronic state change of a fixed structure. In the former case, reorganization of chiral nanostructures has to take place either via photochemical reactions [50] or optical force manipulation [79], thus, the time that required to switch the chiroptic response by light is usually long, which can expands from seconds to minutes, depending on the kinetics of photochemical reactions and complexity of the chiral system. For instance, R. Wang, et al. established a nanorod dimer system with L‐/D‐cysteine linkers [139]. In the P_L_ state, the Au nanorod dimers appear LH under internal torque of the L‐cysteine. When a LCP light is applied to irradiate the solution, a relative torque is applied on the Au nanorod dimer, which rotates the Au nanorods into M_D_ configurations (upper panel in Figure 8a), switching the chiroptic response from positive to negative, which is fully reversible when the CPL light is turned off (bottom panel of Figure 8a). However, as this system is in dynamic colloidal system where lots of thermal fluctuation takes place, it takes ∼40 min to fully revert a cycle of chiroptic switching. Recently, they revealed that the asymmetric photothermal effect is crucial to activate such switching behavior as only temperature above the threshold can weaken the molecular linkages, facilitating selectively chiral switching driven by asymmetric optical torques [140].

Another type of light‐induced structural change is based on light‐responsive liquid crystals (LCs) [141, 142], which mostly involves the mechanism of photoisomerization. Common photoresponsive isomer classes include azobenzene, stilbenes, spiropyrans, diarylethenes, etc [143, 144]. When combined with chiroptic component such as Au nanorod dimers, they can also switch the CD response of the system due to the *trans*–*cis* photoisomerization of azobenzene molecules [145]. However, the major issue is many photoswitchable molecules degrade after hundreds or thousands of cycles, far below the millions required for practical devices. Recently, Zheng and coworkers designed and synthesized intrinsic chiral diarylethene photoswitches to enable digital photoprogramming of multi‐stable LC superstructures with reversible, selectable reflection states across the visible‐NIR spectrum. Such chiral switches show stereospecific photoisomerization (>99% ee), large helical twisting power and high thermal stability. Normally, as the pitch length of LC increases with isomerization, the LC reflection red shifts and the whole switching process takes about 17 s from purple to NIR, which can switch back again after the same period of time (Figure 8b) [146]. Compared with rigid plasmonic or dielectric chiral nanostructures, they offer large‐area processability, reversible optical tunability, direct visual readout, and compatibility with display architectures. Thus, photoresponsive chiral liquid crystals (CLCs) complement light‐induced chiral nanostructures by extending chiral optical functions from nanoscale plasmonic and photonic elements to soft, reconfigurable, and display‐compatible materials.

Light‐induced electronic change of chiral structures is much faster in operation speed, which is highly compatible for chiral optoelectronic devices. Zhang and coworkers designed a 3D chiral metasurfaces on silicon‐on‐sapphire substrate, whose chiroptic response can be tuned by photoexcitation [147]. The ellipticity of the metamolecule monolayer flips and rotates the polarization angle of light in excess of 10° without changing their structure chirality (Figure 8c). Such in situ photo‐induced chiral switching is highly promising for dynamically tunable terahertz circular polarizers and polarization modulators for terahertz radiations.

More recently, Cai's group have demonstrated an achiral dual‐layer metasurface that can be transformed into a handedness‐selectable chiral medium via spatially asymmetric hot‐electron excitation induced by linearly polarized pump light, enabling sub‐picosecond chirality control (Figure 8d) [148]. This hot‐electron‐induced symmetry breaking mechanism has brought up huge advantage of complete chiral inversion simply via handedness‐selectable pump polarization in sub‐picosecond time scale, which has significantly implications for ultrafast chiroptic computation.

## 4 Applications of Light‐Induced Chiral Nanostructures

Due to the facileness of light‐induced chirality, it has been an emerging technique for scalable fabrication of chiral plasmonic nanostructures including helicoids and metasurfaces, which have been applied for chiral biosensing, chiral luminescence devices and biomedical applications [24]. The application of light‐induced chiral nanostructures should be evaluated according to whether the fabrication method satisfies the requirements of the target function. Chiral biosensing prioritizes local optical chirality enhancement, surface accessibility, and low intrinsic background. Chiral luminescence requires spatial and spectral overlap between emitters and chiral modes. Biomedical applications require scalable synthesis, colloidal stability, and biological compatibility. Active polarization optics requires reversible or ultrafast modulation with low insertion loss. Therefore, the following applications are discussed not only as demonstrations of chiroptical performance, but also as examples of how specific light‐induced fabrication and tuning mechanisms address different engineering requirements.

**FIGURE 9:**
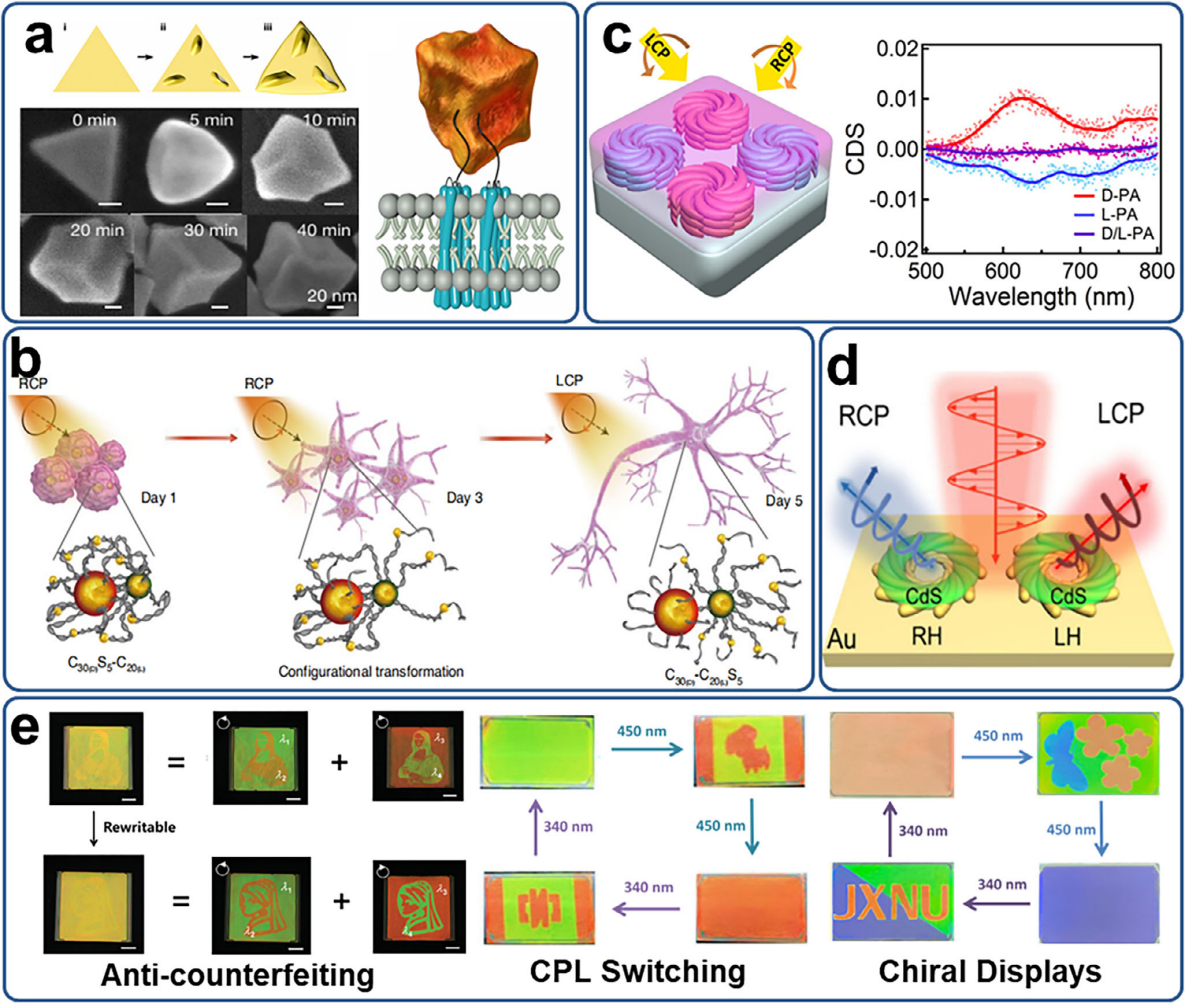
Applications of light‐induced chiral nanostructures. (a) Photosynthesized chiral Au NPs. Left: Schematic of Au deposition stages on nanoprisms under right‐circularly polarized light (top) and SEM images (bottom); Right: Schematic of chirality‐dependent cellular uptake via receptor interactions. Reproduced with permission [150]. Copyright 2022, Springer Nature. (b) Schematic of NSC differentiation under CPL illumination. Reproduced with permission [151]. Copyright 2020, Springer Nature. (c) Schematic of plasmonic racemic chiral biosensing and CDS sensing of D‐, L‐phenylalanine and racemic solutions. Reproduced with permission [152]. Copyright 2024, American Chemical Society. (d) Schematic of CdS emitters located at the hotspots of Au spiral metasurfaces, which give strong CPL. Reproduced with permission [128]. Copyright 2025, American Chemical Society. (e) Applications of CLCs for anti‐counterfeiting (left), CPL switching (middle) and chiral displays (right). Reproduced with permission [154, 155, 156]. Copyright 2024, Wiley‐VCH GmbH, Chinese Chemical Society, and The Royal Society of Chemistry.

Chemical synthesis of chiral NPs with the assistance of chiral ligands or template has been recognized as a scalable route toward chiroptic devices [25]. This is largely due to the chiral transfer from the chiral ligands to nanocrystals based on the oriented surface adsorption [149]. However, their chiroptic response is normally weak, which significantly limits their performance. Aided by CPL, the chiral growth can be further amplified as they selectively facilitate the growth kinetics along the chiral bias endowed by the chiral ligands, which leads to pronounced chiroptic performance with g‐factor up to 0.4 (left panel in Figure 9a) [150]. Kuang and coworkers further utilize these batch‐synthesized chiral NPs to investigate their interactions with biological system (right panel in Figure 9a). They found both in vivo and in vitro immune responses depend monotonically on the g‐factors of the NPs, suggesting a nanoscale chirality pathway for immunology. Not only CPL can be useful for augmenting the chiroptic response to intervene the biological process, it can also promote the differentiation of neural stem cells into neurons with the assistance of DNA‐bridged chiral assemblies of Au NPs (Figure 9b) [151]. This is mainly because the chiral Au NP assemblies can exert forces on the cytoskeleton with matching CPL polarization, which induces periodic mechanical deformation of actin nanofibers, thereby stimulating the differentiation of neural stem cells into the neuronal phenotype. These results clearly manifest the unique advantages of CPL in biomedical applications.

Besides, the light‐induced chiral nanostructures exhibit advanced chiral biosensing capability as they can form racemic arrays by scanning the laser across the substrate (left panel in Figure 9c). These racemic arrays show no intrinsic CD response but bear strong local optical chirality enhancement for chiral analytes [152]. As such, the detected CD signals only reflect the chiroptic response of the chiral biomolecules, which are transferred from UV to visible (right panel in Figure 9c). The limit of detection can reach 10 µm, which is more than an order of magnitude lower than conventional CD spectroscopy. It dispenses with the need for high‐cost fabrication techniques like EBL [153], but acquires high optical chirality enhancement due to the small gaps between the spiral arms in the metastructures.

Another advantage of light‐induced chirality is that it can region‐selectively address emitters in the chiral metastructures so that the maximum chiral light‐matter interaction can be achieved for high performance chiral luminescence. This is realized by polarization‐dependent photochemistry at the hotspots of chiral plasmonic nanocavity, which allows selectively deposition of CdS nanoemitters in the hotspots of the spiral metastructures (Figure 9d) [128]. The spatial and frequency overlap between the emitters and chiral hotspot is critical to maximize the luminescence dissymmetry (g_lum_) via chiral Purcell effect [125]. As a result, the g_lum_ can reach up to 0.5 in the visible region. Moreover, the g_lum_ of the spiral plasmonic metastructures can be tailorable with the polarization orientation of the vector beam as well as the duration of the irradiation [134], which brings many degrees of freedom for adjustable chiroptic devices.

In addition to plasmonic biosensing and chiral luminescence devices, photoresponsive CLCs constitute a practical soft‐matter platform for light‐induced and light‐modulated chirality. Their self‐organized helical superstructures give rise to photonic bandgaps and handedness‐selective reflection, while embedded photoswitchable chiral dopants allow molecular photoisomerization to be converted into changes in helical pitch, reflection wavelength, optical activity, and circular‐polarization response. These CLC materials can be patterned and functional for optical anti‐counterfeiting and information encryption [154], reversible CPL switching [155], and chiral photonic displays (Figure 9e) [156], suggesting that CLCs are not merely model systems for studying dynamic chirality, but are directly relevant to device‐oriented chiral photonics.

## 5 Conclusion and Outlook

Light‐induced chirality has emerged as one of the most promising route for scalable fabrication of chiral nanostructures in recent years, offering unique advantages in direct patterning, dynamic control, and material versatility. The diversity of optical approaches, from direct writing to vortex beams, provides a rich toolkit for creating chiral nanostructures across multiple length scales and material systems.

Recent breakthroughs, particularly in combining CPL with laser direct writing for large‐scale arrays [52, 53], demonstrate that light‐induced methods can achieve the strong chiroptic responses required for practical applications while maintaining the scalability needed for commercialization. This massive 3D fabrication can be further improved with parallel beam systems combined with roll‐to‐roll process [157], which can also be extended to 4D with a responsive material system [158, 159, 160].

A broader range of materials is expected to be sculpted into chiral nanostructures using light. Although the available material platforms have expanded from polymers to metals and semiconductors, light‐induced chirality remains limited to a small number of photosensitive precursors. Developing new photosensitive material systems is therefore essential for extending this approach to a wider range of compositions and functionalities. Alternatively, we can first make chiral arrays with CPL writing and then do in situ reaction such as ion exchange or template replication such as electroplating/evaporation to extend the material systems [161, 162]. With this template approach, functional materials such as hybrid organic‐inorganic materials [163, 164], self‐healing materials [165, 166], hydrogels [167], phase change materials [168, 169], and light‐driven actuators [170, 171] can potentially be coupled to chirality for chiral responsive systems [172].

**FIGURE 10:**
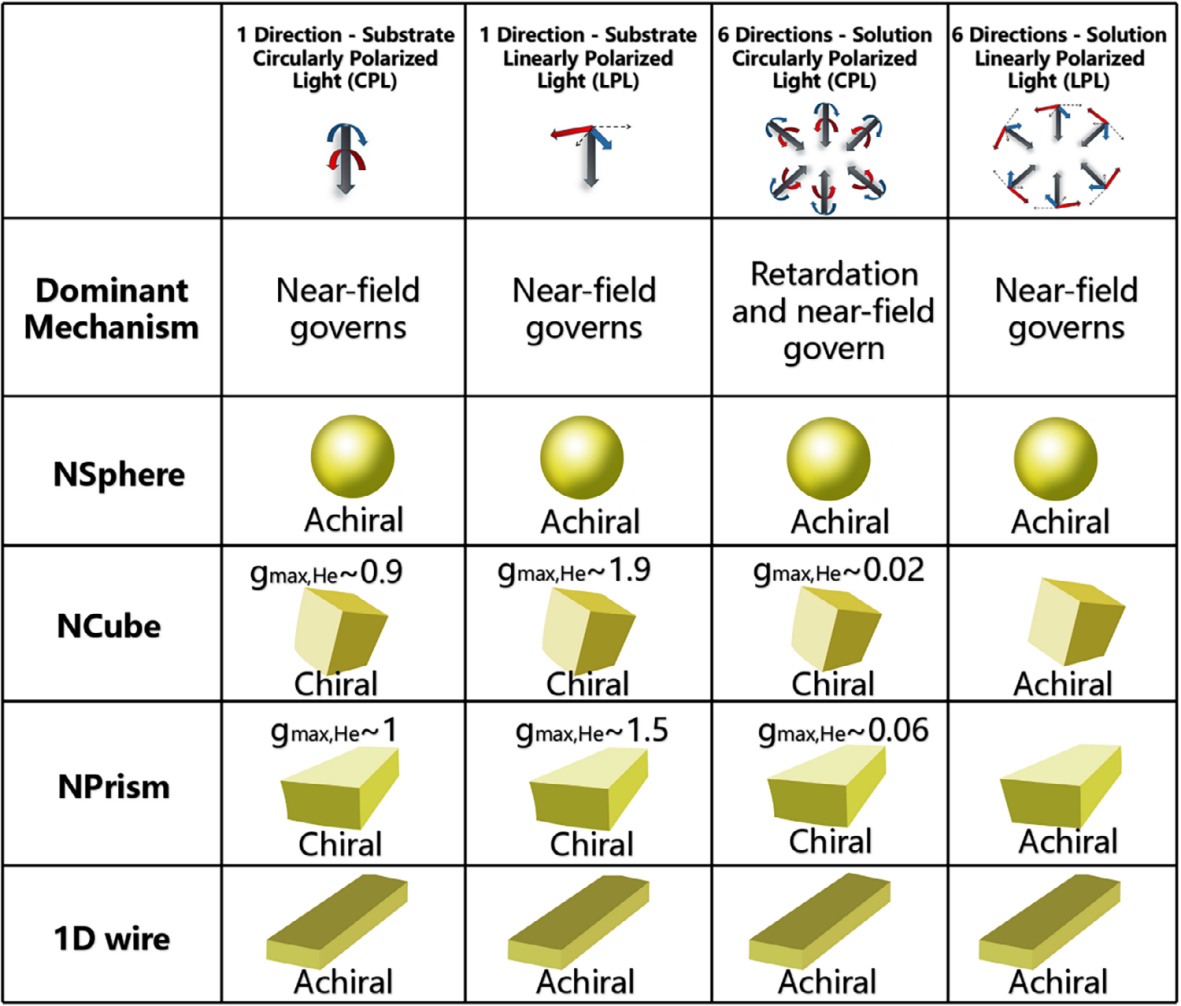
Summary of photoinduced chirality under different illumination condition. The chiral‐achiral appearances reflect the joint symmetry of NC and time‐varying electromagnetic field [174].

The question of how light transfers its chirality to matter should be clarified, especially on the quantum aspects of chirality transfer [173]. Currently understandings are largely phenomenological without analytical expressions, which strongly limits the technological development. To date, various routes for induced chirality in initially achiral nanocrystals (NCs) using polarized light, based on hot‐electron (hot‐e) mechanism are summarized in Figure 10 [174]. For the thermoelectric (T‐mechanism), the table would require adjustments, as the T‐effect typically creates less pronounced chirality because temperature diffusion tends to dissipate the chiral patterns. Chiral patterns fundamentally emerge when the NC geometry and the electric‐field vector together form a chiral arrangement. 2D chirality is readily achieved when an anisotropic NC is under unidirectional illumination (CPL or LPL), yielding highly effective g‐factors (≈1). In contrast, 3D chirality in solution is inherently weaker, originating from retardation effects and diminishing significantly for smaller NCs. Still, CPL can imprint 3D chirality under suitable conditions, producing measurable but smaller g‐factors (typically requiring six‐directional illumination). A hybrid substrate‐solution approach offers an alternative way as NCs grown on substrates under one‐directional illumination acquire z‐asymmetry and can then be released into solution to reveal 3D chirality, though this route faces challenges with low yield and higher costs. Finally, specific NC geometries, like prisms and cubes, are particularly effective because their edges and apexes host intense chiral hot spots. These findings collectively demonstrate that polarized light can effectively and optically induce chirality in plasmonic NCs, enabling promising applications in bio‐related fields.

As light‐induced chirality is evolving from laboratory curiosity to practical technology, the key milestones ahead include commercialization of chiral metasurface products, integration with silicon photonics, biomedical applications, chiral single photons for quantum technologies, among others. We believe with better theoretical understanding, expanded material compatibility, and improved process control, light‐induced chirality will transition from an emerging technique to a cornerstone technology for next‐generation photonic devices, sensors, and quantum systems. The future of chiral nanomaterials is bright, which is literally illuminated by the light that creates them!

## Conflicts of Interest

The authors declare no conflicts of interest.

## Data Availability

No data was generated in this research.
